# Nanoscale organization of the MHC I peptide-loading complex in human dendritic cells

**DOI:** 10.1007/s00018-022-04472-2

**Published:** 2022-08-10

**Authors:** Nicole Koller, Philipp Höllthaler, Martina Barends, Marius Döring, Christoph Spahn, Verónica Durán, Bibiana Costa, Jennifer Becker, Mike Heilemann, Ulrich Kalinke, Robert Tampé

**Affiliations:** 1 Institute of Biochemistry, Biocenter, Goethe University Frankfurt, Max-von-Laue-Str. 9, 60438 Frankfurt am Main, Germany; 2 Institute for Experimental Infection Research, TWINCORE, Centre for Experimental and Clinical Infection Research, a joint venture between the Helmholtz-Centre for Infection Research and the Hannover Medical School, 30625 Feodor-Lynen-Str. 7, Hannover, Germany; 3 Institute of Physical and Theoretical Chemistry, Goethe University Frankfurt, Max-von-Laue-Str. 9, 60438 Frankfurt am Main, Germany; 4 Cluster of Excellence - Resolving Infection Susceptibility (RESIST), Hannover Medical School, Carl-Neuberg-Straße 1, 30625 Hannover, Germany

**Keywords:** Antigen processing, Membrane organization, Membrane proteins, Nanoscopy, ABC transporter, Super-resolution microscopy

## Abstract

Dendritic cells (DCs) translate local innate immune responses into long-lasting adaptive immunity by priming antigen-specific T cells. Accordingly, there is an ample interest in exploiting DCs for therapeutic purposes, e.g., in personalized immunotherapies. Despite recent advances in elucidating molecular pathways of antigen processing, in DCs the exact spatial organization of the underlying processes is largely unknown. Here, we unraveled the nanoscale organization of the transporter associated with antigen processing (TAP)-dependent peptide-loading machinery in human monocyte-derived DCs (moDC). We detected an unexpected accumulation of MHC I peptide-loading complexes (PLCs) and TAP-dependent peptide compartmentalization in protrusions of activated DCs. Using single-molecule localization microscopy we revealed that PLCs display homogeneously sized assemblies, independent of the DC activation status or cellular localization. Our data indicate that moDCs show augmentation of subcellular PLC density during DC maturation. We observed a twofold density increase in the cell body, while an even fourfold accumulation was detected in the tips of the protrusions at the mature DC stage in comparison to immature DCs. In these tip regions, PLC assemblies are found along highly compressed tubular ER networks. These findings provide novel insights into nanoscale organization of the antigen presentation machinery, and open new perspectives on the T cell stimulatory capacity of DCs.

**Supplementary Information:**

The online version contains supplementary material available at 10.1007/s00018-022-04472-2.

## Introduction

Dendritic cells (DCs) are key regulators of adaptive immunity. They take up and process antigens to display antigenic peptides in complex with major histocompatibility complex (MHC) molecules on their cell surface. In secondary lymphoid organs such as lymph nodes, DCs prime antigen-specific T cells and serve as a rheostat that determines T-cell responses [1, 2]. Due to their antigen presentation capacity, DCs are extensively exploited in anti-cancer immunotherapies as well as in applications for personalized medicine [3, 4]. The heterogeneous population of DCs can be subdivided into monocyte-derived DCs (moDCs), plasmacytoid DCs, Langerhans cells, and conventional DCs, depending on their function, localization, and phenotype [5–7]. moDCs, which are frequently used for therapeutic purposes, are differentiated in vitro from monocytes that can be purified in large numbers from peripheral blood mononuclear cells [4, 8, 9]. In vivo, monocytes are recruited to sites of inflammation, where they locally differentiate to DCs. Immature DCs (imDCs) are characterized by low surface expression of MHC II and co-stimulatory molecules as well as by their limited mobility and high capacity to phagocytose particulate antigens. Upon further stimulation, imDCs develop into mature DCs (mDCs), which are identified by upregulated expression of co-stimulatory molecules, reorganization of the cellular structure, and enhanced motility [10, 11]. The migratory behavior of mDCs is associated with the formation of actin-rich protrusions at the leading edge of the cell and by a passive movement at the trailing edge, which is also referred to as the uropod [12, 13]. All these maturation-associated functional and morphological changes are essential for antigen-specific priming of T cells [10, 11].

One key component of antigen presentation is the ER-resident transporter associated with antigen processing (TAP), consisting of TAP1 and TAP2, which translocates peptides from the cytosol into the ER lumen. TAP is involved in both the canonical MHC I pathway and in the presentation of exogenously acquired antigens on MHC I molecules, a process termed cross-presentation. TAP together with the chaperones tapasin, calreticulin, ERp57, and different MHC I allomorphs forms the macromolecular (650 kDa) peptide-loading complex (PLC) [14]. Once assembled, the PLC orchestrates the loading of peptides onto MHC I molecules, which are exposed on the DC surface to prime antigen-specific CD8^+^ cytotoxic T cells [15–17]. While the role of the PLC is well-described in the canonical MHC I pathway, the extent of its involvement in cross-presentation is still under debate [17–20]. Major findings in the molecular organization of antigen processing and presentation have been described in cell lines. Furthermore, reports studying antigen presentation in the context of immune responses have focused on the capacity of DCs to induce T cell restimulation. To this end, studies on cross-presentation analyzed the capacity to stimulate antigen-specific T cells as a read-out for the efficiency of antigen presentation by DCs [18, 19, 21, 22]. Nevertheless, it remains unclear whether in DCs antigen processing and presentation occur locally at preferred subcellular sites [23, 24], as the central component of the PLC, TAP, localizes to the ER and *cis*-Golgi [25]. However, recent findings about the dynamic morphology of the ER and its complex compartmentalization [26] call for re-evaluation of the subcellular localization of the TAP-dependent antigen-processing machinery. In particular, the subcellular architecture, molecular composition, and function of the PLC in professional antigen-presenting cell subsets such as DCs are poorly resolved. Additional factors such as vesicle trafficking proteins have been identified to be involved in MHC I recruitment to cross-presentation-competent organelles [27–30]. Furthermore, a role for microtubule-directed endosome localization/maturation in cross-presentation was described in DCs [31, 32]. Thus, analyzing the subcellular distribution of the peptide-loading machinery in DCs on the nanoscale level will increase our understanding of antigen presentation pathways.

Here, we delineate the subcellular organization of the endogenous PLC in human moDCs during differentiation and maturation in 2D culture for nanoscale microscopy analysis. To this end, we used a well-established model of moDC differentiation and maturation on microscopy slides that are specifically coated for adherence and growth of fastidious cells [33]. Employing this model, we analyzed the nanoscale organization of the PLC in subcellular compartments of imDCs and mDCs. We discovered that, independent of the DC maturation status, the PLC is organized in nano-structures of a defined size that sequester peptides independent of their subcellular localization. PLC assemblies are embedded in a tubular ER membrane meshwork that spans throughout the DC. Upon maturation, the PLC distribution is altered in the cell body and can even double in its density. In the tips of mDC protrusions, where the ER meshwork is compressed, the PLC density even quadruples. Our study opens new avenues to explore the TAP-dependent direct antigen presentation and cross-presentation that is relevant in tumor control as well as in viral infections.

## Methods

### Isolation of CD14^+^ monocytes from human peripheral blood

Buffy coats were diluted with 1 × Dulbecco’s phosphate-buffered saline (PBS), and PBMCs were isolated by density-based separation through a Ficoll layer (Biocoll, *δ* = 1.077 g/mL, isotonic, Biochrom AG) for 20 min at 900*xg.* PBMCs were washed twice, and CD14^+^ cells were isolated by MACS-positive selection using human-CD14 MicroBeads (Miltenyi). For isolation, manufacturer’s protocols for manual separation using LS columns or automatic separation in the AutoMACS Pro Separator (Miltenyi) were followed. After isolation, cells were resuspended in CellGro serum-free DC medium (CellGenix). Informed consent was obtained from all donors as approved by the Ethics Committee from the DRK-Blutspendedienst Baden-Württemberg/Hessen and Blutspendedienst NSTOB in Springe, Germany. Subject data were treated as confidential information protected by medical confidentiality.

### Differentiation from monocytes to moDCs

Procedure was followed as previously described [33]. In short, isolated monocytes were differentiated over a period of five days to imDCs by the addition of interleukin (IL)-4 and granulocyte–macrophage stimulating factor (CellGenix, 1000 Units/mL each) in Nunc® Lab-Tek® II Chamber Slide™ system. For further maturation, imDCs were stimulated with 10 ng/mL tumor necrosis factor (TNF) α (PeproTech), 1000 U/mL IL-6 (PeproTech), 10 ng/ml IL-1β (PeproTech), and 1 mg/mL prostaglandin *E*_2_ (Cayman).

### Lentiviral vector particle production and monocyte transduction

HEK 293 T cells were transfected with a three vector system consisting of pViFCGdBH, sgp.d2, and pMD.G2. Lentiviral vector production and titration was performed as described [34]. Monocytes were lentivirally transduced with TAP1 C-terminally fused with mVenus. The next day, cells were differentiated and matured to mDCs as described above.

### Co-immunoprecipitation

Monocyte, imDC, and mDC pellets stored at − 80 °C (~ 1.0–1.5 × 10^7^ cells per cell type per condition) were thawed on ice and lysed for 1 h in co-IP buffer (150 mM NaCl, 50 mM HEPES pH 7.4, 8.6% (*v/v*) glycerin) supplemented with 1% (*w*/*v*) digitonin, 2.5 mM benzamidine, and 1 mM phenylmethylsulfonyl fluoride (PMSF). To avoid donor-to-donor variances, a pool of two to three different donors were sampled. For co-immunoprecipitation, 50 μg/condition Dynabeads™ M-280 sheep-anti-mouse IgG (Thermo Fisher Scientific) were incubated with 0.1% (*w*/*v*) BSA/PBS for 15 min at RT. Subsequently, 10 μg/condition α-TAP1 (mAb 148.3) or mouse IgG_1_, *κ* isotype antibody (abcam) were added. Upon incubation for 2 h at 4 °C, the antibodies were crosslinked with 13.0 mg/ml dimethyl pimelimidate (Pierce™ DMP cross-linker, Thermo Fisher Scientific) in 0.2 M sodium borate pH 8.8 for 20 min at RT. Beads were washed with 0.2 M triethanolamine/PBS, and the crosslinking step was repeated for three times in total. At the end, beads were washed with 1 M Tris/HCl pH 8.9 and stored in PBS at 4 °C until usage. Upon cell lysis, samples were centrifuged for 30 min at 100,000x*g* at 4 °C and solubilizates were precleared with 25 ug/condition Dynabeads™ for 30 min at 4 °C with overhead rotation. Next, solubilizates were added to α-TAP1 antibody- or isotype antibody-bound Dynabeads™ and incubated for 3 h at 4 °C with head-over-tail rotator. Beads were washed in Co-IP buffer with 0.1% (*w*/*v*) digitonin. Proteins were eluted in 50 μl 2 × oxidizing SDS buffer at 65 °C 300 rpm for 10 min. β-mercaptoethanol was added to the eluate afterwards to a final concentration of 0.7 M. Aliquot samples of solubilizate were resuspended in 1 × reducing SDS-buffer. All samples were stored at − 20 °C.

### Immunoblotting

Cells were lysed on ice for 1 h in buffer containing 1% (*w*/*v*) digitonin, 150 mM NaCl, 50 mM HEPES pH 7.4, and 8.6% (*v*/*v*) glycerin. Samples were centrifuged for 30 min at 4 °C at 100,000x*g*, resuspended in reducing SDS sample buffer, and consequently incubated with 1% benzonase (Merck) for 15 min at room temperature (RT). The samples were subsequently denatured at 65 °C for 10 min, separated by 10% Tris–glycine gels, and blotted onto polyvinylidene fluoride membranes. Samples from TAP1 co-immunoprecipitation were thawed, heated for 10 min at 65 °C, and separated by SDS-PAGE. Membranes were blocked with 5% milk in TBS-T buffer prior to incubation with primary antibody: mouse α-TAP1 (1:10, hybridoma, clone 148.3), rat α-tapasin (1:3000, clone 7F6-1–1), mouse-α-MHC I (1:200, hybridoma, clone HC10), rabbit α-calreticulin (1:2000, abcam or Sigma-Aldrich), rabbit α-ERp57 (1:2000, abcam), rabbit α-Sec61α (1:2000, abcam) for 2 h at RT or overnight at 4 °C. Subsequently, membranes were incubated with secondary antibodies α-goat, α-mouse, α-rat, or α-rabbit horseradish peroxidase (HRP, Merck/Sigma) for 1 h at RT. For GAPDH, the membrane was incubated with α-GAPDH-HRP (1:2000, Biolegend) for 1 h at RT. Membranes were developed with Clarity Western ECL Reagent (BioRad) at Lumi™ F1 system (Roche) or Fusion FX (Vilber).

### Flow cytometry

Cells were stained in FACS buffer (2% (*w*/*v*) BSA, 20 mM EDTA, and 0.2% (*w*/*v*) sodium azide in PBS, and mixed with 10% (*v*/*v*) polyglobin (Gamunex) for FcγR blocking for 20 min at 4 °C. Consequently, antibodies were added and incubated for 20 min at 4 °C. Cells were washed in FACS buffer and resuspended in 0.25% (*v*/*v*) formaldehyde/FACS buffer. Samples were acquired with the BD FACSCelesta™ (Becton Dickinson (BD)) using the instrument specific software. Data analysis was performed with FlowJo™ V10 (Treestar, San Carlos, CA). The following fluorophore-coupled antibodies were used: fluorescein-5-isothiocyanate (FITC) *α*-human-MHC II (BD), FITC mouse IgG2a, *κ* isotype (BD), allophycocyanin (APC) *α*-human-CD86 (Biolegend), APC mouse IgG2b, *κ* (Biolegend), brilliant violet 421 (BV421) *α*-human-CD83 (Biolegend), BV421 mouse IgG1, *κ* (Biolegend), phycoerythrin (PE) *α*-human-CD14 (Biolegend), and PE Mouse IgG2a, *κ* (Biolegend).

### Immunofluorescence

Upon isolation, monocytes were seeded in 8-well chamber slides with a chemically coated growth surface on glass slides that mimics polylysine (Nunc Lab-Tek II, Thermo Fisher Scientific) and incubated for 30 min at 37 °C. Cells were washed in PBS before fixing with 3% (*v*/*v*) formaldehyde/PBS for 15–20 min at RT. After quenching with 50 mM glycine (Roth)/PBS for 10 min, monocytes were permeabilized with 0.1% (*v*/*v*) Triton X-100 (Sigma) in PBS for 20 min. Unspecific binding was blocked with 5% (*w*/*v*) BSA/PBS for 2 h at RT, before incubating with mouse α-TAP1 (1.5 µg/mL, monoclonal, clone 148.3) in 1% (*w*/*v*) BSA/PBS overnight at 4 °C. After several washing steps with PBS, secondary antibody (1:1000, final 2 µg/mL, polyclonal, Thermo Fisher Scientific) was added for 1 h at RT in the dark. For nuclei visualization, cells were incubated for 2 min with 4,6-diamidino-2-phenylindole (DAPI) (Thermo Fisher Scientific). After several PBS washing steps, monocytes were fixed in 3% (*v*/*v*) formaldehyde/PBS and mounted with fluorescent mounting medium (DAKO). For moDCs, monocytes were differentiated and matured directly in 8-well chamber slides and fixed with 3% (*v*/*v*) formaldehyde/PBS for 15–20 min. Cells were blocked and permeabilized with 3% (w/v) BSA, 1.5% (*w*/*v*) glycine, and 0.01% (*w*/*v*) saponin in PBS for 1 h at RT. Consequently, cells were washed and stained with the same antibodies as for monocytes but in 0.1% (*w*/*v*) BSA and 0.01% (*w*/*v*) saponin in PBS. Secondary antibody goat α-mouse^AF568^ or donkey α-mouse^AF488^ were used. Cells were mounted with fluorescence mounting medium (DAKO) imaged at Airy Scan LSM880 (Zeiss) with Plan-Apochromat 63x/1.4 oil objective (Olympus).

For dual-color immunofluorescence staining, cells were fixed in PHEM buffer (60 mM PIPES, 25 mM HEPES, 10 mM EGTA, 4 mM MgSO_4_, adjusted to pH 6.9 with KOH) containing 3% (*v*/*v*) formaldehyde and 0.1% (*v/v*) glutaraldehyde for 1 h at RT. After quenching with freshly prepared 0.2% (*w*/*v*) sodium borohydride in PBS for 7 min, staining protocol for moDCs was followed. After incubation with primary antibodies mouse α-TAP1 and rabbit α-Calnexin (1:200, final 5 µg/mL, monoclonal, Abcam) overnight at 4 °C, secondary antibodies goat α-rabbit^AF532^ (1:1000, final 2 µg/mL, polyclonal, Thermo Fisher Scientific) and goat α-mouse^AF647^ (1:1000, final 2 µg/mL, Thermo Fisher Scientific) were applied for 1 h at RT in the dark. Upon addition of fluorescence mounting medium (DAKO), samples were imaged at LSM880 with airy scan detector in SR-mode.

For single-molecule localization microscopy analysis of TAP1 single staining, moDCs were washed extensively with PBS after incubation with primary antibody mouse-*α*-TAP1 (if not otherwise indicated with 1.5 µg/mL) and secondary antibody goat *α*-mouse^AF647^(if not otherwise indicated 1:100, final 20 µg/mL, Thermo Fisher Scientific), fixed in 3% (*v*/*v*) formaldehyde for 10 min, and stored in PBS at 4 °C until imaging. For dual-color analysis, moDCs staining protocol was followed as mentioned above and incubated with secondary antibodies goat α-rabbit^AF532^ (1:100, final 20 µg/mL, polyclonal, Thermo Fisher Scientific) and goat *α*-mouse^AF647^ (1:100, final 20 µg/mL, Thermo Fisher Scientific) for 1 h at RT in the dark.

For Nile Red staining, monocytes and moDCs were fixed in 8-well chamber slides using 3% (*v*/*v*) formaldehyde/PBS for 45 min at RT. Nile Red (Thermo Fisher Scientific) was added at a concentration of 0.3 µM in PBS. Imaging was performed at LSM880 (Zeiss) with excitation laser 532 nm, applying the airy scan detector in SR-mode.

### Single cell-based translocation assay

mDCs were matured in 8-well chamber slides as for immunofluorescence staining. Protocol was adapted from previous publication [35]. Cells were semi-permeabilized for 15 min at 4 °C with ~ 0.5–0.8 µg/mL streptolysin-O (Abcam), and transport was carried out in 30 nM RRYQNSTC^AF647^L in the presence of 10 mM ATP or ADP, and 10 mM MgCl_2_ in PBS. Cells were incubated for 30 min at 37 °C and then imaged with excitation laser 633 nm at LSM880 (Zeiss) with Plan-Apochromat 63x/1.4 oil objective (Olympus). Peptide translocation in TAP1-mVenus expressing mDCs was imaged with C PL APO CS2 40x/1.10 water objective (Leica) at 37 °C and 5% CO_2_ with SP8 lightning microscope (Leica) using excitation lasers 514 nm and 633 nm for mVenus and Alexa Fluor 647, respectively.

### Single-molecule localization microscopy

Super-resolution images were recorded using the *d*STORM protocol [36]. NanoImager (Oxford Nano Imaging) was used for imaging of DCs. Immunolabeled samples were imaged upon excitation with a 640 nm laser using UPLSAPO 100x/1.4 oil objective (Olympus) for long acquisition times, a focus lock maintains a stable z-position over time using an infrared laser. Illumination angles were adjusted from wide-field to HILO. Emitted fluorescence signal was separated from excitation light with a 640 nm long pass dichroic mirror. The red channel was additionally filtered with a band pass filter (685/40) ensuring a high signal-to-background ratio. 30,000 frames were collected on a Flash 4 V3 sCMOS (Hamamatsu, 121 nm pixel size) with 30 ms integration time and an automated stepwise increase of the 405 nm laser intensity every 10,000 frames for single-color *d*STORM to recover the fluorophores from the dark state and to control emitter density for robust single-molecule imaging. Fluorophores were excited with 640 nm at an illumination density of 3 kW/cm^2^. Samples were kept in a freshly prepared TRIS/MEA-buffer (pH 8.0, 100 mM TRIS, 100 mM 2-mercaptoethylamine (MEA)) at 30 °C using the integrated heating stage. Stable microscope temperature ensured minimal drift of the sample in xy directions. Additionally, dual-color *d*STORM experiments of TAP1 (Alexa Fluor 647) and Calnexin (Alexa Fluor 532) were performed. The red channel (TAP1) was recorded as described for single-color experiments except that the samples were first illuminated with 640 nm without additional 405 nm exposure for 30,000 frames. After read-out of the TAP1 signal, 532 nm illumination (~ 3 kW/cm^2^) was used for the green channel for an additional 30,000 frames. Green signal was filtered with a second band pass filter (585/70) and 0.1 µm Tetraspecks (Invitrogen) were added as a fiducial marker for drift correction and channel alignment.

### Image and data analysis

*d*STORM raw data were analyzed using the Picasso software package [37]. Single point spread functions (PSF) were fitted with the “Localize” function of Picasso utilizing an image gradient (net-gradient 5000) and yielded the *x*,*y*-position of single fluorophores. Additional analysis steps in Picasso included drift correction (redundant cross correlation with a window of 1000 frames or fiducial markers for dual-color images), PSF shape filtering (using a sigma range for the wide-field PSF between 48.4 and 193.6 nm) and spatiotemporal linking of single-molecule emission events (72.6 nm (representing 6 times the experimental localization precision of 12.1 nm) and two dark frames). This resulted in reconstructed images of 5–13 cells for each donor including control and isotype measurements.

The filtered localization lists were further analyzed with Picasso DBSCAN plugin to identify TAP1 signals and extract the diameter and number of detected single-molecule events for each signal. Of note, robust DBSCAN analysis requires a sophisticated choice of the required parameters, namely the observation radius and the number of localizations that must be found within this radius to form a cluster object (min_pts) [38]. We extracted these parameters from each individual single-molecule measurement to exclude artifacts arising from variation in photoswitching dynamics. The observation radius was set as a 2 × of the experimental localization precision, determined from a nearest-neighbor analysis (typically in the range of 22–35 nm (radius) and 20–80 events as minimum threshold for clusters) [39]. To determine min_pts, 50–100 clusters were manually annotated, and clusters with similar properties were automatically detected by the Picasso function “Pick similar” with a 1.5 × standard deviation. min_pts was then determined as the median of the number of localizations per signal minus 1 × the median absolute deviation (MAD). Signal information extracted by DBSCAN analysis was visualized with Picasso and statistically analyzed with OriginPro 9 (OriginLab Cooperation, USA) for each condition and depicted as mean values with their respective standard errors in box plots.

The cluster density map was generated where a grayscale value was assigned to the center of mass (COM) of each cluster with LocAlization Microscopy Analyzer (LAMA) [40] Afterwards, reconstructed *d*STORM images were used to determine the outline of each dendritic cell using ImageJ [41] and a custom-written macro. The area of the previously determined cell outline and the integrated signal of all COMs (i.e., the number of signals) for each cell were measured in ImageJ and further analyzed in OriginPro 9. Statistical analysis was performed with GraphPad Prism V8 (GraphPad, La Jolla, CA) using the Kruskal–Wallis-test with Dunn’s correction for multiple comparisons.

## Results

### Molecular composition of the PLC during DC differentiation and maturation

**Fig. 1:**
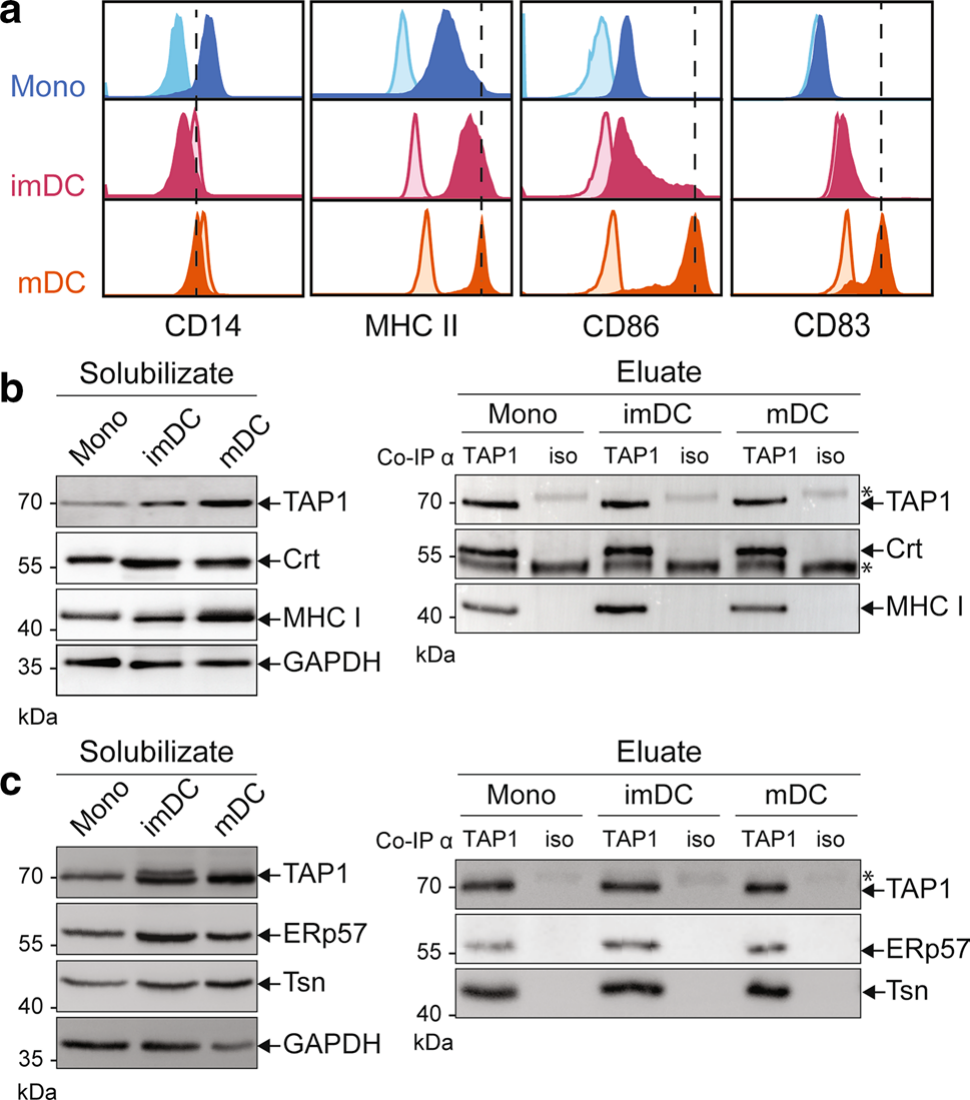
moDCs differentiation and TAP1 co-immunoprecipitation of the PLC. **a** Expression of the surface markers CD14, MHC II, CD86, and CD83 during differentiation from monocytes (Mono, blue) to imDCs (pink) and maturation to mDCs (orange). Isotypes are shown in corresponding light-colored histograms. **b** Monocytes (Mono), imDCs, and mDCs were solubilized in 1% (w/v) digitonin and co-immunoprecipitated with a TAP1-specific monoclonal antibody (TAP1) or a corresponding isotype control antibody (iso). Left panel shows cell lysate (solubilizate), and right panel displays co-IP fractions of (**b**) TAP1, Crt (calreticulin), and MHC I as well as (**c**) TAP1, ERp57, and Tsn (tapasin) immunoblots. GAPDH served as a loading control. *Corresponds to IgG chains of anti-TAP1 and isotype antibodies

To characterize differentiation and maturation of monocytes into imDCs and mDCs, we analyzed the expression of marker proteins by flow cytometry and determined changes in cell morphology. To this end, we added a cytokine cocktail that is commonly used in clinical studies for DC maturation [42]. During DC differentiation, the expression of the monocytic marker CD14 decreased, while MHC II and the activation marker CD86 were upregulated (Fig. 1a). In addition, during DC maturation, CD83 was enhanced (Fig. 1a). Along these changes, TAP1 expression increased (Fig. 1b, left panel). Primary cells (monocytes, imDCs and mDCs) were further subjected to TAP1 co-immunoprecipitation (co-IP) followed by immunoblot analysis to discover possible differences in molecular composition of the PLC. An isotype control was included to probe for unspecific binding. Directly after cell lysis (solubilizate), all PLC components were detected with slight variations in their expression levels between monocytes, imDCs and mDCs, when compared with the loading control GAPDH (Fig. 1b and c, left panels). For comparison of the PLC composition, the TAP1 amount was kept constant in the eluate of the different cell types. No major differences were noticed in the amount of tapasin, ERp57, calreticulin, and MHC I associated with TAP in monocytes, imDCs, and mDCs. PLC components were not detected in the control co-IPs (Fig. 1b and c, right panels). In addition, the control ER-marker Sec61α did not co-elute with the PLC (Supplementary Fig. 1). Thus, the decoration of TAP1 with antibody was specific and indicated an overall conserved composition of the main PLC components during primary cell differentiation.

### Changes in subcellular PLC distribution during DC differentiation and maturation

**Fig. 2:**
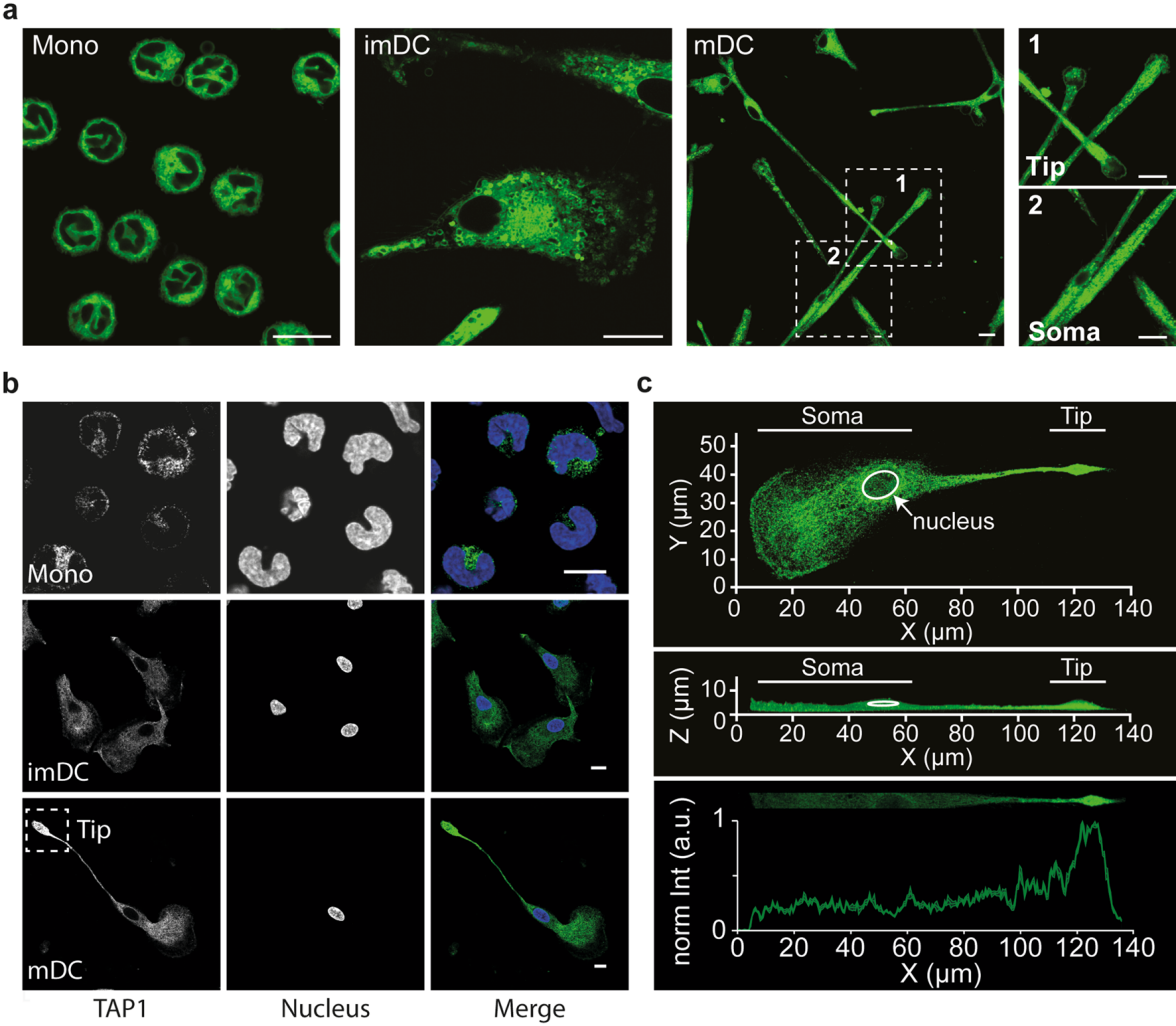
moDCs undergo morphological changes that are accompanied by PLC redistribution. Monocytes (Mono), imDC and mDC were imaged by confocal microscopy. **a** Cell membrane stained by the lipophilic dye Nile Red in fixed monocytes, imDCs, and mDCs. Scale bar, 10 µm. **b** Immunofluorescence staining of TAP in monocytes, imDCs, and mDCs. Scale bar, 10 µm. **c** Representative TAP distribution in mDCs, imaged by confocal laser-scanning microscopy (CLSM) in z-stacks, shown in length (x-axis), width (y-axis), and height (z-axis). TAP distribution in mDCs is highlighted by the intensity profile of TAP1 staining derived from ImageJ (plot profile tool)

We next addressed the subcellular localization of the PLC in DCs. Since TAP is the central component of the PLC, we used TAP1 as a marker for this multi-subunit translocation and chaperone machinery. During DC differentiation, the morphology of small circular-shaped monocytes flattened and elongated at the stage of imDCs (Fig. 2a). In conclusion, we were able to confirm that this model, which previously was applied to analyze the subcellular PLC distribution in monocytes and moDCs [33], allows an in situ differentiation of monocytes to homogenous imDC cultures and subsequent maturation to mDC cultures without the need to resuspend and transfer the cells to microscopy slides for further microscopic analysis. Here, we examined the organization of the PLC in DCs at the nanoscale level using super-resolution microscopy. We observed that in this cell model, mDCs further extended and formed elongated protrusions, which is an indicator for enhanced motility. To investigate the subcellular organization of the PLC, we subdivided mDCs into two regions, (i) the peripheral tip region, which in motile DCs sometimes is referred to as the trailing edge, and (ii) the perinuclear soma region, which can also be referred to as the leading edge (Fig. 2a, dashed squares 1 and 2, respectively). The analysis of subcellular structures in mDCs revealed densely packed intracellular membranes in the soma and the tip region of the mDC protrusions (Fig. 2a). TAP1 immunofluorescence labeling demonstrated that in monocytes, the PLCs were present in distinct signals (Fig. 2b, top panel). Interestingly, in imDCs, the PLCs were scattered throughout the cell, whereas in mDCs, the PLCs accumulated at the tip region of mDC protrusions (Fig. 2b, middle and lower panel, Fig. 2c and Supplementary Fig. 2a). Using the common ER-marker calnexin, we showed that not only the PLC but also the ER accumulated in the tip region (Supplementary Fig. 2b, c). Thus, we conclude that the protrusions of mDCs contain enhanced density of PLCs, particularly in their tips.

### The PLC sequesters peptides in both soma and tip regions of dendritic cells

**Fig. 3:**
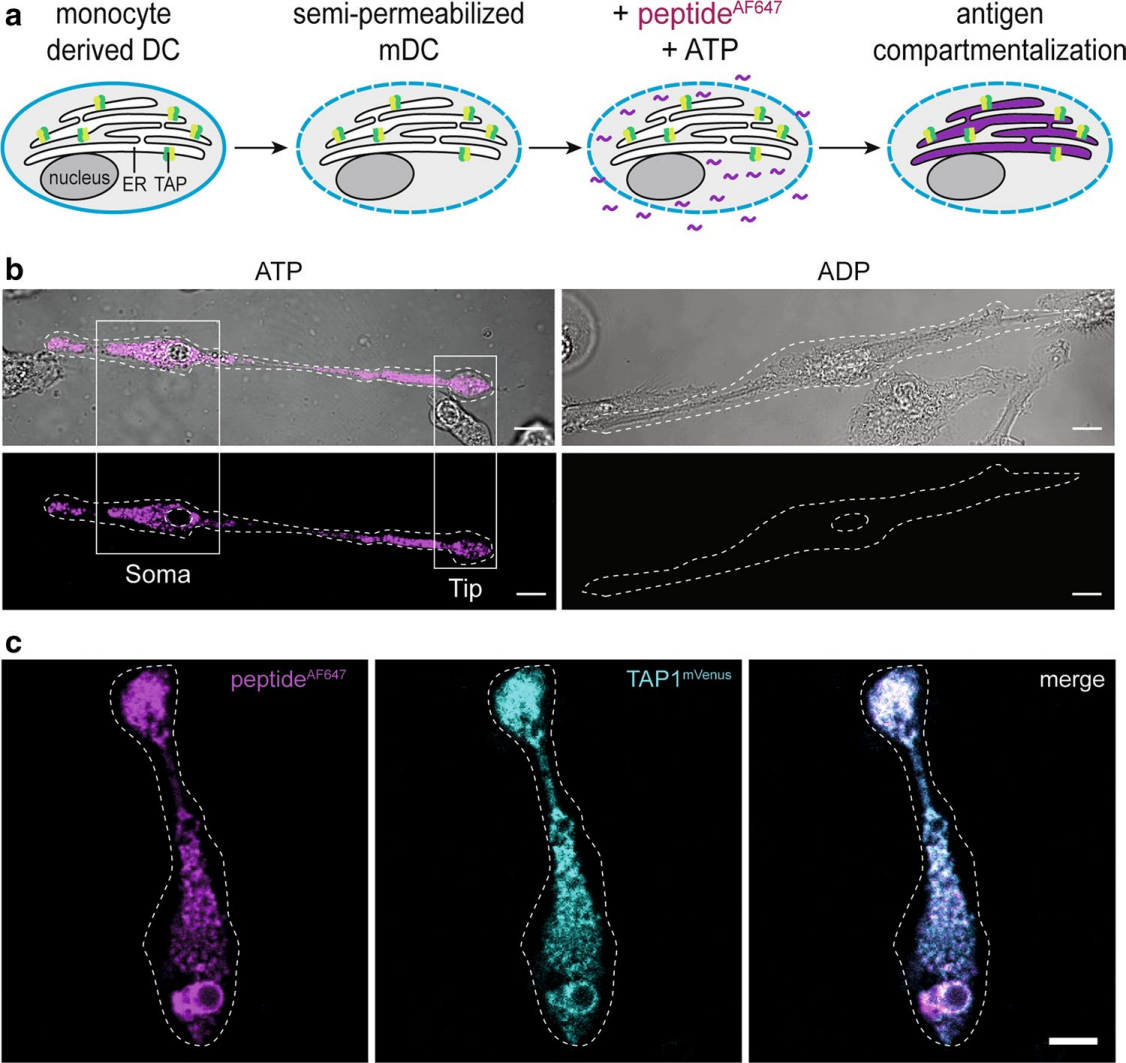
The PLC is active in both soma and dendritic tips of mDCs. **a** Schematic workflow of the TAP-dependent peptide translocation assay. Plasma membrane was semi-permeabilized by streptolysin-O while the ER membrane stayed intact. Upon addition of 30 nM peptide labeled with Alexa Fluor 647 (peptide^AF647^) in the presence of ATP or ADP, cells were incubated at 37 °C. During incubation, TAP sequesters peptide^AF647^ into the ER lumen. **b** mDCs were semi-permeabilized and peptide^AF647^ (magenta) accumulation was imaged in the presence of ATP or ADP. Scale bar, 10 µm. **c** TAP-dependent peptide (magenta) accumulation with TAP1^mVenus^ (cyan) expressing mDCs in the presence of ATP. Due to the very complex organization of the tubular ER, the peptide signal cannot directly be correlated with the number of local TAP/PLC complexes. Scale bar, 10 µm

To address whether cytosolic peptides are compartmentalized by locally active TAP in the perinuclear ER or the ER of the tip region, we established a live-cell translocation assay of antigenic peptides monitored by confocal laser-scanning microscopy (CLSM). The plasma membrane of mDCs was semi-permeabilized by the *Streptococcus*-derived toxin streptolysin-O, while keeping ER membranes intact (Fig. 3a). The ER integrity was previously verified in cell lines and primary cells by the loss of cytosolic GFP signal during semi-permeabilization, while the ER-resident KDEL-mCherry signal was preserved [33, 35]. ER-resident TAP transported fluorescent peptides in an ATP-dependent manner from the cytosol into the ER lumen, where the peptides stayed trapped due to *N*-core glycosylation [35, 43]. We have further shown that peptide accumulation occurs not only in an ATP- but also TAP-dependent manner [33, 35]. In the presence of ATP, we observed a TAP-dependent antigen sequestration throughout the whole cell, both in soma and tip regions (Fig. 3b), which co-localized with the PLC (Fig. 3c). Thus, the PLC is active in both soma and tip regions of mDCs.

### Nanoscale analysis of the PLC ER-distribution in dendritic cells

**Fig. 4:**
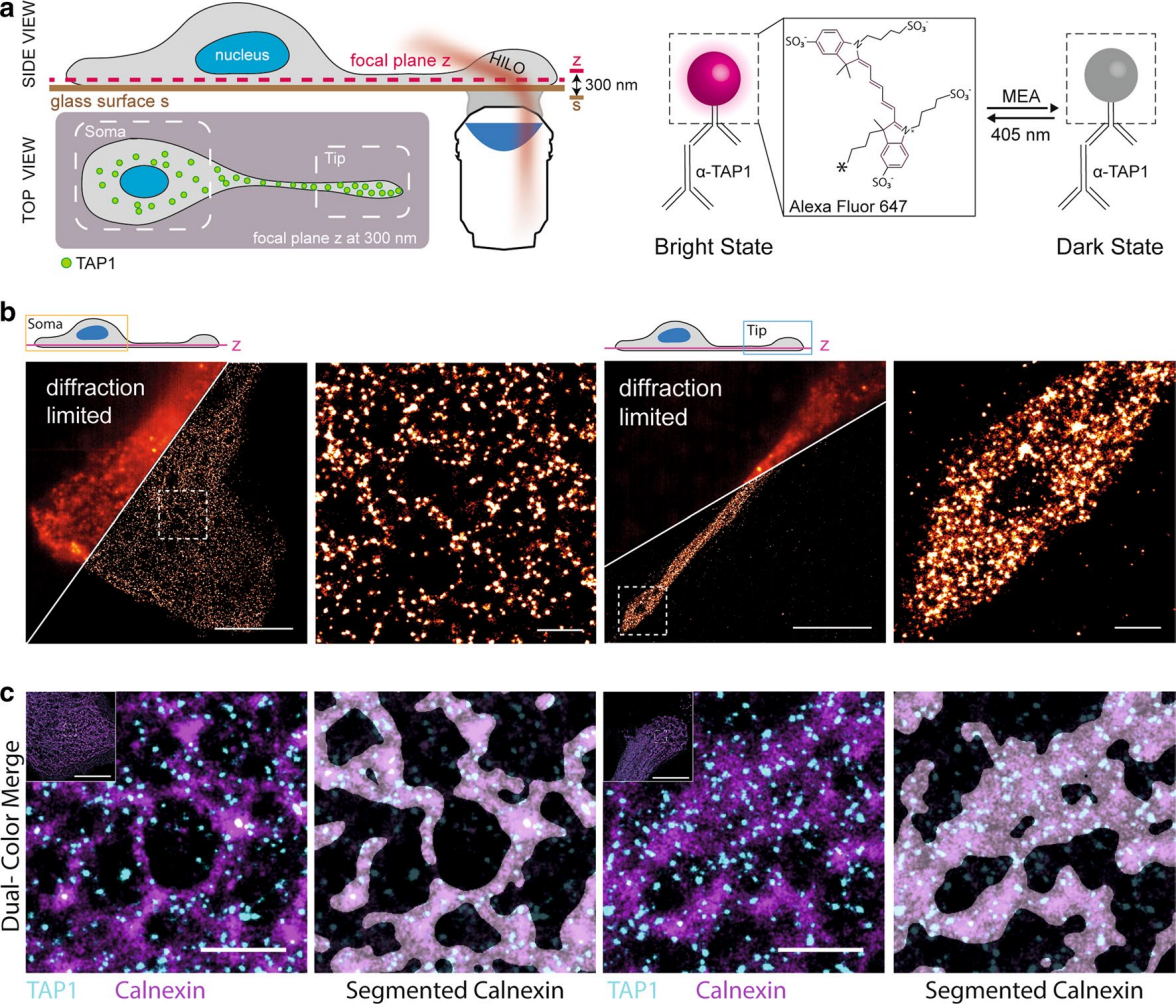
Tubular ER structures harboring PLCs are confined in the tips of mDC extensions. **a** Schematic side and top view of a DC for SMLM imaging. Organization of PLC was visualized by immunolabeled TAP1 molecules using highly inclined and laminated optical sheet (HILO) illumination mode to ensure fluorophore excitation at a defined focal plane (300 nm above the glass surface). Dyes were photoswitched between a dark OFF-state and a bright, light-emitting ON-state providing a subset of fluorescent molecules in the light-emitting state for imaging. Number of blinking events was controlled by an increasing dose of 405 nm illumination. **b** Comparison of diffraction-limited immunofluorescence with reconstructed super-resolution images of TAP1 in mDCs. Zoom-in images show TAP1 signal distribution in the soma (left panel) and the tip region (right panel). Scale bars are 10 µm for overview images and 1 µm for zoom-ins. **c** Dual-color super-resolution image of TAP1 (cyan) and the ER-marker protein calnexin (magenta). Scale bar, 10 µm for overview images and 1 µm for zoom-ins. Calnexin shows distinct structures of the ER leaflets with TAP localized in close range to the soma region (left panel). Highly condensed localizations of TAP1 signal in mDC tip shows unstructured regions for both proteins (right panel). Segmented view of calnexin is shown to further highlight the proximity with TAP

To address whether the PLC accumulation in the tips of mDC protrusions was due to an enhancement of local PLC density, we studied the subcellular localization of the PLC at the nanoscale. Single-molecule localization microscopy (SMLM) reaches 20 nm resolution, which is tenfold higher than diffraction-limited microscopy [36, 44]. To improve the signal-to-background ratio, moDCs were imaged in highly inclined and laminated optical sheet (HILO) mode, for which a 2D culture system with adherent cells is essential. HILO imaging further improves the optical section and minimizes projection effects, allowing analysis of the TAP distributions in different cellular subregions (e.g., peripheral and perinuclear regions). For reasons of comparability, imaging was performed at a focal plane 300 nm above the glass surface (Fig. 4a). While in diffraction-limited microscopy the PLC distribution in imDCs and mDCs appeared blurry and with varying signal density and structure (Fig. 4b and Supplementary Fig. 3), SMLM revealed individual and spatially separated signals of the PLC in the soma region (Fig. 4b, left). In addition, SMLM images showed an increased density of PLC assemblies in the tips of mDC protrusions (Fig. 4c, right), which was consistent with the higher signal of fluorescent peptides observed in the live-cell translocation assay (Fig. 3c). We also applied dual-color SMLM to image the PLCs in relation to the architecture of the ER labeled by the ER-marker calnexin. In the soma region, tubular ER structures were observed that were highly condensed in the tips of mDC protrusions. We showed that the majority of PLCs are ER-resident, with some TAP1 signals outside of calnexin-positive compartments (Fig. 4c and Supplementary Fig. 4). These data are consistent with previous studies in imDCs and mDCs that showed a recruitment of TAP1 to LAMP1-positive compartments, in addition to the well-described ER localization [33], and underline that the PLC resides in highly compressed tubular ER structures in the tips of mDC protrusions.

### PLC density increases during DC maturation

**Fig. 5:**
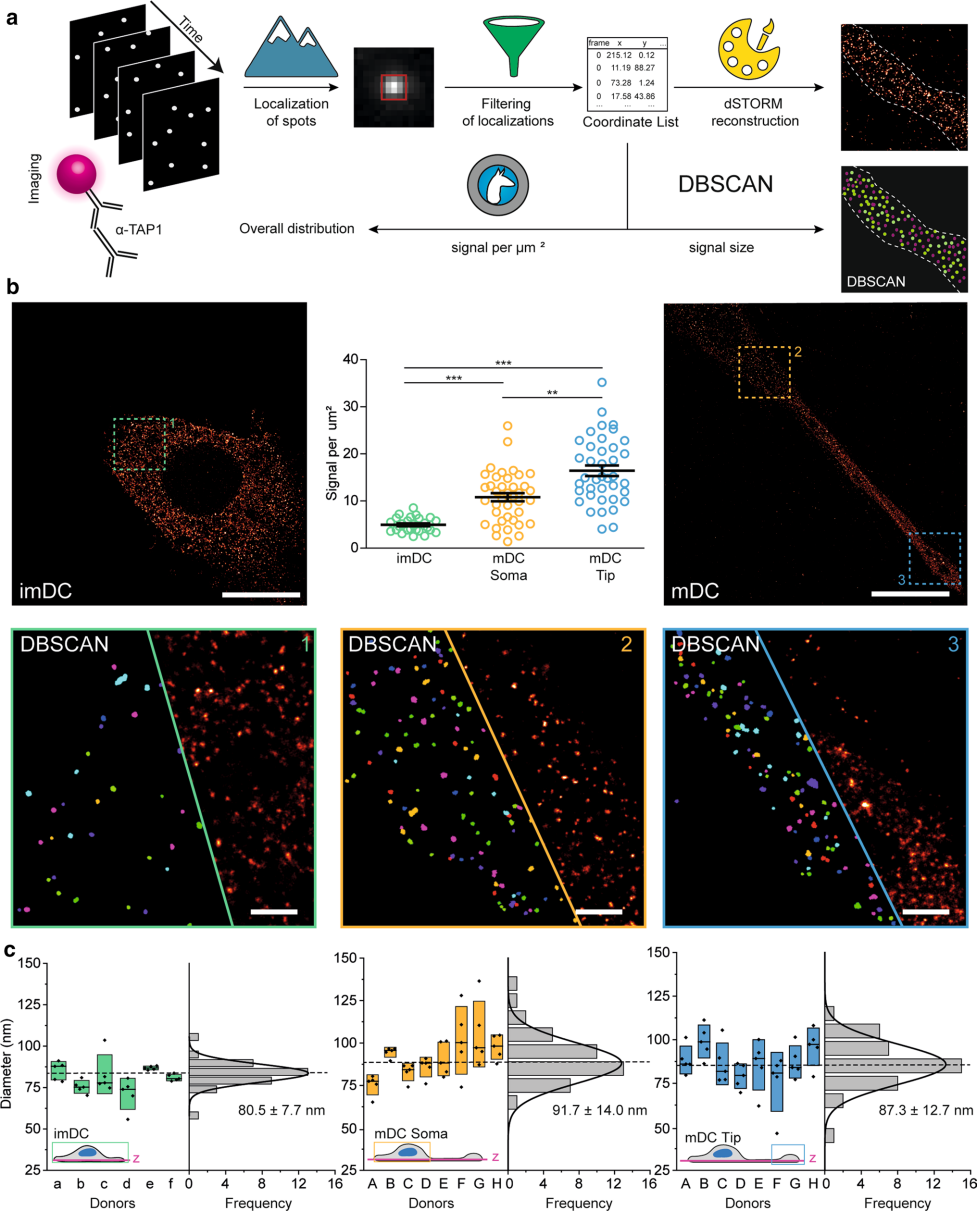
PLC density increases upon DC maturation. **a** Data acquisition and analysis workflow for signal analysis in moDCs. Localization of single-molecule signals followed by filtering the datasets and rendering (reconstruction) provides a super-resolved image of each cell. Using density-based spatial clustering of applications with noise (DBSCAN), implemented in the Picasso software, enables determination of localization signal size followed by counting of localization signals per cell using the analysis tool LocAlization Microscopy Analyzer (LAMA). **b** Images were analyzed for the signal density (signals/µm^2^). Top left panel depicts an overview of a reconstructed image of an imDC and top right panel a mDC comprising soma and tip region. Each image is split in half illustrating DBSCAN signals and super-resolved image. Representative examples for visualization of TAP signals are depicted with close-ups of (1) imDC with least signals per area, (2) mDC soma region exhibiting a higher density, and (3) mDC tip region showing an outmost dense distribution. Scale bar overview, 10 µm and close-up, 1 µm. Top middle panel summarizes number of signals per area for imDC with six donors and mDCs soma and tip with eight donors. Average density of 4.9 ± 0.3 signals per µm^2^ were found in imDCs, in the soma of mDCs 10.8 ± 0.9 signals per µm^2^ (yellow), and in the tip 17.0 ± 1.3 signals per µm^2^ (blue). Means ± SEM are shown. **P value ≤ 0.01, ***P value ≤ 0.001, Kruskal–Wallis-test with Dunn’s correction for multiple comparisons was used for statistical analysis. **c** Average signal diameter for imDC (six donors a-f, *n* = 6) was 80.5 ± 7.7 nm and for mDC soma region 91.7 ± 14.0 nm and tip region 87.3 ± 12.7 nm (eight donors A-H, *n* = 8). Per individual donor, median diameter of five individual imaged cells were analyzed. Values are visualized as floating bars and averaged diameters are determined by Gaussian fit function

We investigated the spatial distribution of PLC assemblies in moDCs using a classical immunofluorescence labeling with primary and secondary antibodies. We assessed the PLC density in imDCs as well as in soma or tip regions of mDCs using a density-based cluster analysis (DBSCAN) (Fig. 5a and Supplementary Fig. 5) [38]. Highest PLC density was found in the tip region of mDC protrusions with 17.0 ± 1.3 signals per µm^2^ (Fig. 5b), where also compartmentalization of antigenic peptide was detected (Fig. 3b). In comparison, soma mDC regions showed 10.8 ± 0.9 signals per µm^2^, while imDCs exhibited the lowest density with only 4.9 ± 0.3 signals per µm^2^ (Fig. 5b). Of note, we only detected negligible signals in the isotype control both in soma and tip regions (Supplementary Fig. 6), excluding that the increased signal density is a result of increased stickiness of antibodies in crowded cellular environments. Distance calculations revealed that in the soma region PLC signals were ~ 600 nm apart, while in the tip region the distance was ~ 300 nm, reflecting a quadruplication in density. These results indicate that in DCs PLCs display a heterogeneous distribution, with an increase in density during maturation, depending on the subcellular localization analyzed.

For imDCs and mDCs, the PLC signal size was in the range of 80–90 nm for cells from many different donors, regardless of the cellular localization in the soma or the tip region (Fig. 5c). The overestimation of the diameter due to primary and secondary antibody labeling (two times 10 nm), as well as the uncertainty of the single-molecule localization (i.e., the localization precision of 11–13 nm), are not considered in this value. Interestingly, despite increased TAP protein levels in mDCs (Fig. 1b), the size of PLC assemblies did not increase upon DC maturation (Fig. 5c). We further verified that the PLC labeling was saturated and that unspecific binding of the primary or secondary antibody was neglectable (Supplementary Figs. 7 and 8). Furthermore, different fixation methods did not affect the size of the PLC assemblies (Supplementary Fig. 9). These results indicate a conserved diameter throughout donors and maturation states suggesting that optimal biological PLC assembly is achieved at approximately 90 nm, without high variances. In conclusion, DCs respond to inflammatory stimuli by an increase in PLC density, while the particular signal diameter stays constant.

## Discussion

T-cell priming is a complex and highly regulated process that is of key relevance during the initiation of adaptive immunity. This process requires efficient antigen compartmentalization and processing by professional antigen-presenting cells. By applying single-molecule localization microscopy (SMLM), we uncovered that, during DC differentiation and maturation, DCs form long protrusions that show increased numbers of PLC assemblies especially in their tips, which also contain densely packed ER structures. We found that in DCs the vast majority of these PLC assemblies is embedded in the tubular ER meshwork. Moreover, we describe nanoscale details of the organization of TAP-containing PLCs in moDCs. In addition, enhanced TAP expression was neither associated with an increase in the size of the PLC assemblies nor with the formation of higher-order structures (on the scale of several hundred nanometers and above) during the maturation process. Instead, the density of the PLC assemblies increased during imDC to mDC maturation and was most pronounced in the tips of mDC protrusions. These observations are in accordance with our finding that peptides are accumulated in PLC-dense ER regions. Thus, we provide evidence for the accumulation of PLCs upon maturation in the tips of human DC protrusions.

We were able to confirm that during moDC differentiation and maturation, not only the expression of cell surface markers changes, but also TAP protein levels increase and peak at the mDC stage; however, PLC molecular composition remains unaltered. These changes have been described to be associated with adaptations of moDCs to become potent stimulators of T-cell responses [10, 11]. The in situ differentiation protocol used in this study leads to adherence of moDCs and is known to modulate DC motility [45]. The adherent moDCs are required for the analysis of subcellular compartments, such as the ER, using high-power fluorescence microscopy. Under these culturing conditions, we defined the morphological structures as mDC protrusions, the trailing edge, and the fanned-out soma region, leading edge. One biological advantage of this elongated morphology, besides the migration to lymph nodes, could be to increase the T-cell–APC contact surface [46]. We revealed that the tip regions of mDC protrusions harbor a tightly packed tubular ER meshwork, which comprises active TAP for antigen compartmentalization. In this model system, the DC morphology may resemble that of monocyte-derived macrophages. However, the surface marker expression of the moDC generated in this system indicates their homogeneity [47].

By mapping and analyzing the PLC distribution in moDCs at nanometer resolution, we found that the PLCs are particularly enriched in the tips of mDC protrusions. Indeed, we detected a defined ER network in the soma region of mDCs that is reminiscent of previously described tubular ER matrices [26]. Using different super-resolution imaging techniques, similar ER structures have recently been identified in the fibroblast-like cell line COS-7 [48]. In the tips of mDC protrusions, the ER tubules were extremely tightly packed so that we could trace only parts of the tubular ER meshwork. This implies that the ER and PLC are actively confined in the tips of these protrusions. Whether this effect is restricted to the tip of mDC protrusions, or may also be found in mDC dendrites, as detected in 3D systems, needs to be addressed in future studies. It is conceivable that in the periphery of DCs, the condensed ER with embedded PLCs at high density might be strategic to promote T-cell activation more readily. As reported for human and mouse cells [49–51], specific peptide-loaded MHC I molecules are organized in clusters at the cell surface. Correspondingly, increased PLC density would facilitate the local loading and later generation of such clusters, thereby controlling T-cell sensitivity [50]. Future studies analyzing DC/T-cell interaction should take this into consideration and should investigate if there are preferred subcellular sites for this interaction.

The surprisingly overall defined size range of PLC assemblies across different cell subsets, regardless of TAP protein level or cell differentiation state, supports the hypothesis that the size of such supramolecular PLC structures has a defined margin [14, 52]. It is conceivable that the size of these PLC assemblies reflects an evolutionarily ideal supramolecular arrangement and that fine-tuning of its function is controlled rather by accumulation of PLC assemblies at a particular subcellular localization than by the absolute size of single PLC assemblies [37]. It remains to be analyzed whether additional factors are required to control or are able to disrupt the subcellular organization of the PLC. In this study, we report for the first time on the nanoscale organization of endogenous PLCs in differentiated non-immortalized human immune cells.

This work aimed at the nanoscale analysis of the PLC, centered around the TAP complex, during the maturation of human moDCs. Super-resolution microscopy allowed the estimation of the size and density of PLC assemblies. The unique distribution pattern of the PLCs and their particularly enhanced density in the tips of cellular protrusions highlight that the supramolecular organization of the TAP-dependent antigen processing machinery is more elaborate than previously assumed. Thus, our findings open new perspectives to further elucidate the molecular mechanisms of the TAP-dependent antigen presentation pathway. Understanding the regulation of cross-presentation by DCs is of key relevance for control of tumor development and viral infections.

### Supplementary Information

The online version contains Supplementary material available at https://doi.XXX.

Supplementary file1 (XLS 36 KB)

Supplementary file2 (PDF 51 KB)

Supplementary file3 (PDF 1926 KB)

Supplementary file4 (PDF 14165 KB)

## Data Availability

All data are available from public resources.
