# Supplementary material for: Nanoscale organization of the MHC I peptide-loading complex in human dendritic cells
Source: Cell Mol Life Sci. 2022 Aug 10;79(9):477. doi: 10.1007/s00018-022-04472-2 (PMC9365725; doi:10.1007/s00018-022-04472-2)
Supplement: Supplementary file 2 — Supplementary file2 (PDF 51 KB) [file 18_2022_4472_MOESM2_ESM.pdf]

Figure 1a

	P1/Single Cells | Median (Comp-APC-A) (	P1/Single Cells | Median (Comp-BV421-A) (	P1/Single Cells | Median (Comp-FITC-A) N	P1/Single Cells | Median (Comp-PE-A) CD14
Mono D33_Mono_002.fcs	711	241	5075	684
imDC D33_imDc_006.fcs	1007	1155	21964	146
mDC D33_imDc_010.fcs	43455	10477	42023	342
Mono D33_iso_001.fcs	123	191	300	94.4
imDC D33_iso_005.fcs	171	731	663	289
mDC D33_iso_009.fcs	270	1235	1285	507

Figure 5b

imDC TAP1	mDC Soma	mDC Tip	
5.139641	25.95189	13.6707	
4.421745	12.88269	8.098657	
4.040068	17.05326	13.28238	
3.651653	7.660874	9.897072	
2.582612		18.45981	
3.856524	7.660874	10.36097	
3.072956	12.94862	20.55882	
4.648353	10.13008	26.171	
3.899808	12.18153	4.421101	
	10.16004	23.29792	
2.467637			
6.589686	15.59166	22.8613	
7.054223	9.930045	26.04541	
5.514806	15.11921	7.64253	
	9.258444	22.81477	
	10.9879	35.18961	
3.9004			
3.910684	13.00428	11.24899	
3.594476	16.06098	19.12556	
	13.22851	21.30139	
	5.155417	20.00191	
6.324244	15.83118		
5.020412			
5.304327	5.008197	24.741	
8.558686	10.55997	12.17919	
5.796423	14.81247	15.20901	
	9.07496	21.55058	
6.401556	16.4963	12.68253	
7.225269			
6.523897	6.609343	11.94345	
3.323471	13.10412	12.69447	
5.78542	4.16933	7.619923	
	5.931672	14.92802	
	2.599812	18.64486	
	22.57339	4.033727	
	15.69999	13.75554	
	11.69566	28.89844	
	12.06427	14.56243	
	11.70518	9.836858	
	1.36993	25.29813	
	2.666364	17.88641	
	3.869605	10.22769	
	5.750804	15.17594	
	4.91198	14.81859	
4.9	10.8	16.4	mean
2.79814652	5.3	6.9	SD
0.30590386	0.91149179	1.30949318	SEM

Figure 5c

diameter (nm) PLC assemblies

imDC

	Donor 10	Donor 13	Donor 16	Donor 17	Donor 18	Donor 19
	Cell	Cell	Cell	Cell	Cell	Cell
cell 1	78.65	75.28	103.58	75.53	87.74	83.72
cell 2	88.37	74.03	77.85	73.91	85.48	82.37
cell 3	79.91	81	77.36	55.8	86.03	79.73
cell 4	87.83	70.29	74.78	80.66	86.78	80.31
cell 5	91.15	76.51	81.58	70.02	88.17	78.91

imDC aver	STDEV	
80.44	8.340941	nm

mDC

	Donor 1				Donor 2		Donor 3		Donor 5		Donor 6		Donor 7		Donor 10		Donor 11	
	Soma	Tip	soma	Tip	soma	Tip	soma	Tip	soma	Tip	soma	Tip	soma	Tip	soma	Tip	soma	Tip
cell 1	78.08	101.26	78.08	101.26	95.57	94.59	84.28	81.81	88.17	79.5	79.96	83.94	110.82	85.12	136.47	77.11	87.74	99.96
cell 2	80.43	79.61	80.43	79.61	89.53	98.74	83.05	77.11	88.27	85.16	83.05	61.97	127.97	80.86	94.99	83.16	93.4	97.31
cell 3	77.48	86.03	77.48	86.03	96.54	111.26	74.16	89.53	85.81	69.62	101.16	100.07	74.06	87.97	97.22	84.05	98.17	78.79
cell 4	75.77	89.01	75.77	89.01	96.54	86.24	86.67	76.87	76.02	86.35	88.27	89.22	100.25	78.68	110.42	101.44	104.61	95.57
cell 5	65.19	85.92	65.19	85.92	94.59	103.98	86.57	105.49	90.87	74.78	100.7	92.5	94.77	46.21	87.21	90.46	104.07	108.11

cell		Tip		
Average al	STDEV	Average al	STDEV	
91.72265	13.96508	87.13462	12.65231	nm

Supplementary Figure 6a

	imDC Donor 10			imDC Donor 13			imDC Donor 16			
	Counts/Area [µm²]			Counts/Area [µm²]			Counts/Area [µm²]			
	Cells	control	Isotype	Cells	control	Isotype	Cells	control	Isotype	
	5.50	0.07	0.11	2.58	0.02	0.24	2.47	0.34	0.32	
	5.14	0.12	0.11	3.86	0.03	0.11	6.59	0.17	0.10	
	4.42	0.10	0.05	3.07	0.24	0.26	7.05		0.10	
	4.04			4.65			5.51		0.12	
	3.65			3.90					0.05	
	4.6	0.10	0.09	3.6	0.10	0.2	5.4	0.3	0.1	average single donors
	0.8	0.02	0.03	0.8	0.12	0.1	2.1	0.1	0.1	standard dev
	cells	control	isotype							
Average all										
	4.5	0.15	0.14							
error propagation:										
	0.78	0.06	0.05							
