# Supplementary material for: Nanoscale organization of the MHC I peptide-loading complex in human dendritic cells
Source: Cell Mol Life Sci. 2022 Aug 10;79(9):477. doi: 10.1007/s00018-022-04472-2 (PMC9365725; doi:10.1007/s00018-022-04472-2)
Supplement: Supplementary file 3 — Supplementary file3 (PDF 1926 KB) [file 18_2022_4472_MOESM3_ESM.pdf]

Supplementary Fig. 1

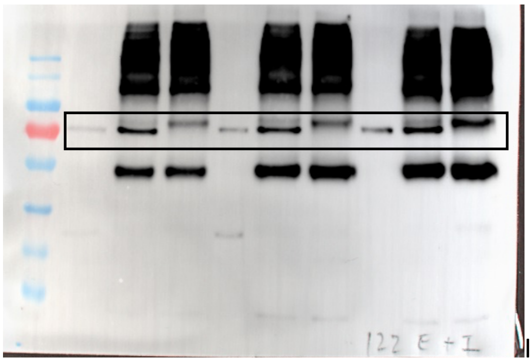

Immunoblot against TAP1

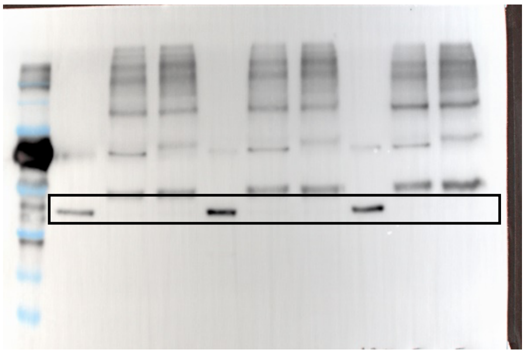

First reblotting of membrane against Sec61α. Unspecific bands are recognized in the marker lane by the Sec61α antibody, as well at around 70 kDa.

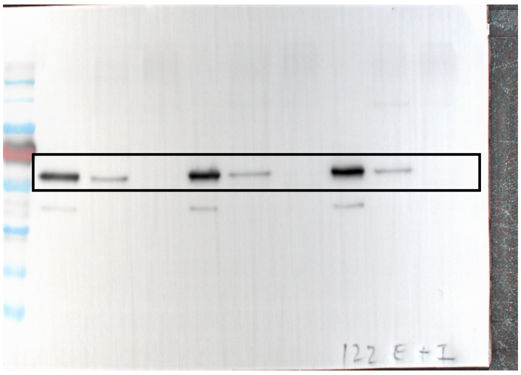

Second reblotting of membrane against calreticulin

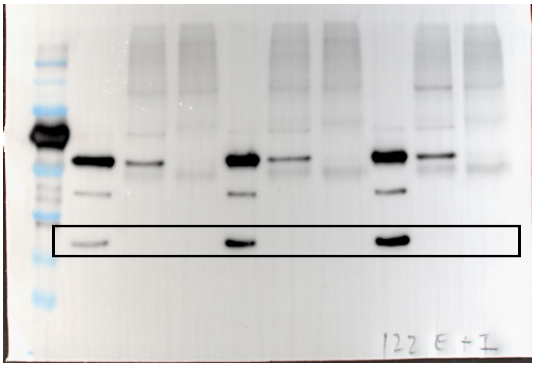

Third reblotting of membrane against GAPDH
