# Supplementary material for: Nanoscale organization of the MHC I peptide-loading complex in human dendritic cells
Source: Cell Mol Life Sci. 2022 Aug 10;79(9):477. doi: 10.1007/s00018-022-04472-2 (PMC9365725; doi:10.1007/s00018-022-04472-2)
Supplement: Supplementary file 4 — Supplementary file4 (PDF 14165 KB) [file 18_2022_4472_MOESM4_ESM.pdf]

Supplemental Information

Nanoscale organization of the MHC I peptide loading complex in human dendritic cells

Nicole Koller[1,#], Philipp Höllthaler[1,#], Martina Barends[1#], Marius Döring[2], Christoph Spahn[3], Verónica Durán[2], Bibiana Costa[2], Jennifer Becker[2], Mike Heilemann[3], Ulrich Kalinke[2,4,*], Robert Tampé[1,*]

[1]Institute of Biochemistry, Biocenter, Goethe University Frankfurt, Max-von-Laue-Str. 9, 60438 Frankfurt am Main, Germany

[2]Institute for Experimental Infection Research, TWINCORE, Centre for Experimental and Clinical Infection Research, a joint venture between the Helmholtz Centre for Infection Research and the Hannover Medical School, 30625 Hannover, Germany

[3]Institute of Physical and Theoretical Chemistry, Goethe University Frankfurt, Max-von-Laue-Str. 9, 60438 Frankfurt am Main, Germany

[4]Cluster of Excellence - Resolving Infection Susceptibility (RESIST), Hannover Medical School, Carl-Neuberg-Straße 1, 30625 Hannover, Germany

[#]contributed equally, *To whom correspondence may be addressed:
Kalinke.Ulrich@mh-hannover.de or tampe@em.uni-frankfurt.de

Supplemenary Figure 1-9

Supplementary Figures

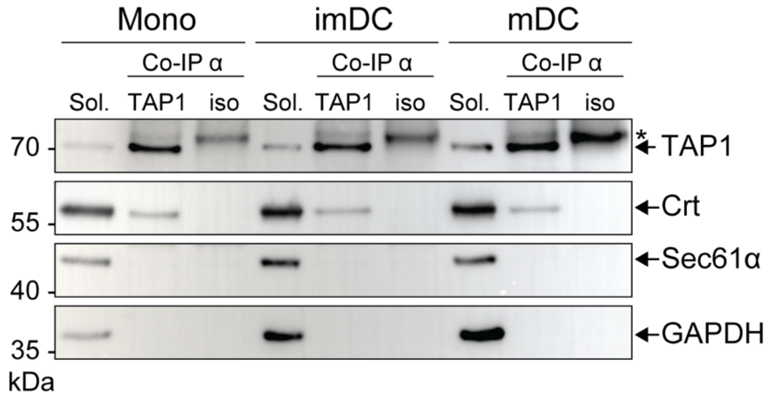

Supplementary Fig. 1. Specific co-elution of TAP1 interacting proteins. Monocytes (Mono), imDCs, and mDCs were solubilized in 1% (w/v) digitonin and co-immunoprecipitated with a TAP1-specific antibody (mAb148.3) or a corresponding isotype control (iso). For every cell type, the first lane shows cell lysate as input (Sol.), followed by the eluate after co-immunoprecipitation (Co-IP α). Samples were analyzed by immunoblotting against TAP1, Crt (calreticulin), Sec61α (ER marker) as negative control, and GAPDH as a loading control for the solubilizate. * IgG from anti-TAP1 or isotype control.

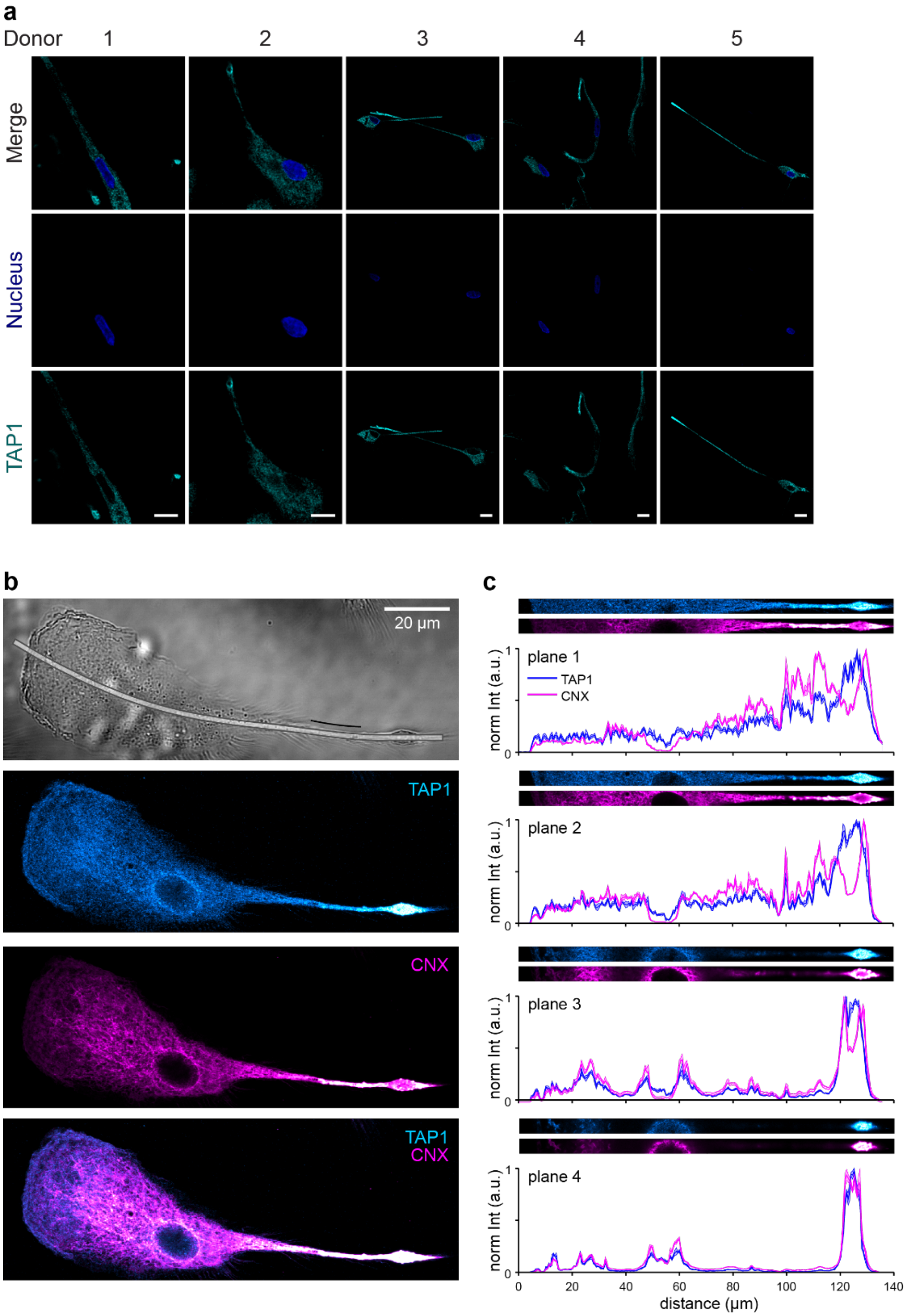

Supplementary Fig. 2. Dual-color confocal laser scanning microscopy of mDCs. a Confocal images of mDCs from five different donors (scale bar 10 µm), IF stained with TAP1 (cyan) and nucleus stained with 1:10 000 Hoechst 33342 in the dark for 2 minutes. **b** Brightfield image (top) and maximum intensity projections of TAP1 and CNX (middle), as well as their overlay (bottom) are shown. (**c**) Exemplary intensity line profiles and their corresponding cell z-images of the white line region shown in the bright field image in (**b**) for different z-planes within the cell are illustrated.

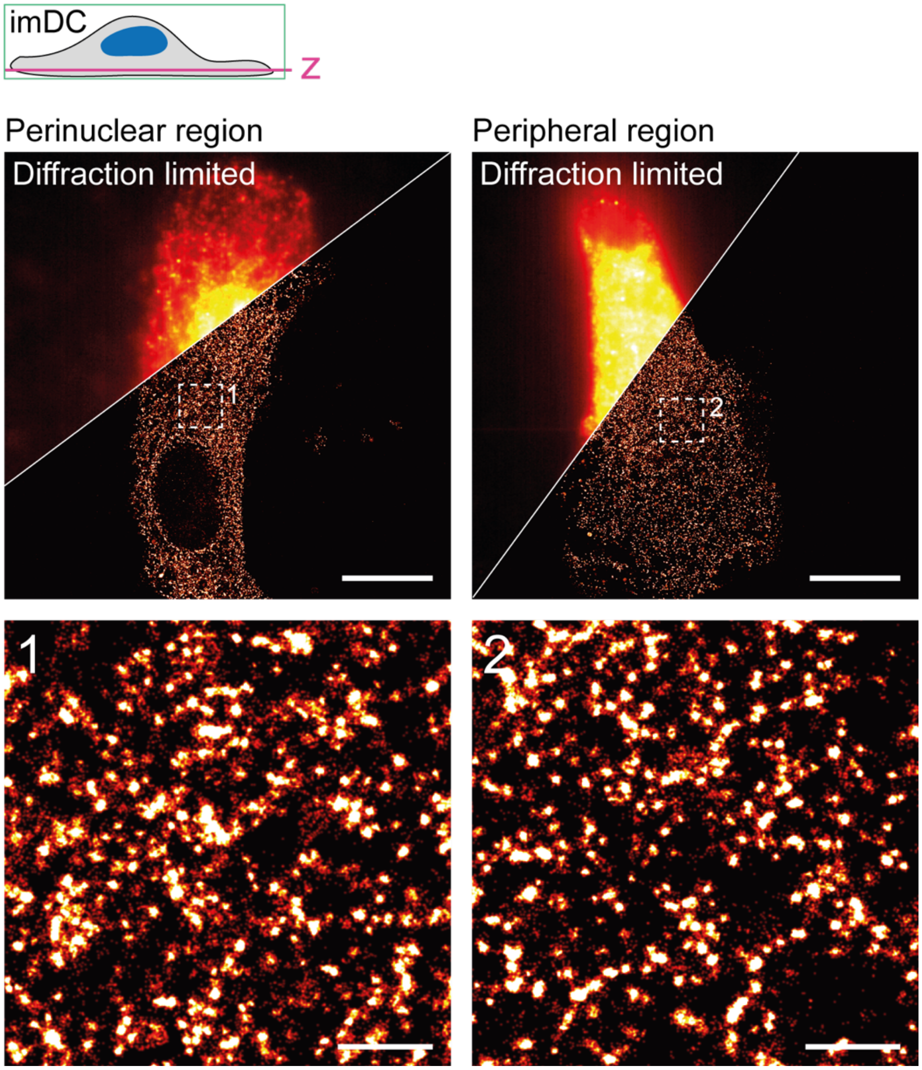

Supplementary Fig. 3. imDCs analyzed by single-molecule localization microscopy (*d*STORM). imDCs were fixed with 3% (v/v) formaldehyde for 1 h and subsequently TAP1 was labeled with primary and AF647-conjugated secondary antibody. Cells were imaged in HILO illumination configuration at an imaging depth of ~300 nm into the cell from the glass surface. The widefield image provided a blurred image lacking distinct patterns of PLC signals. In contrast, the reconstructed super-resolved image exhibits distinct signal structures (lower panel).

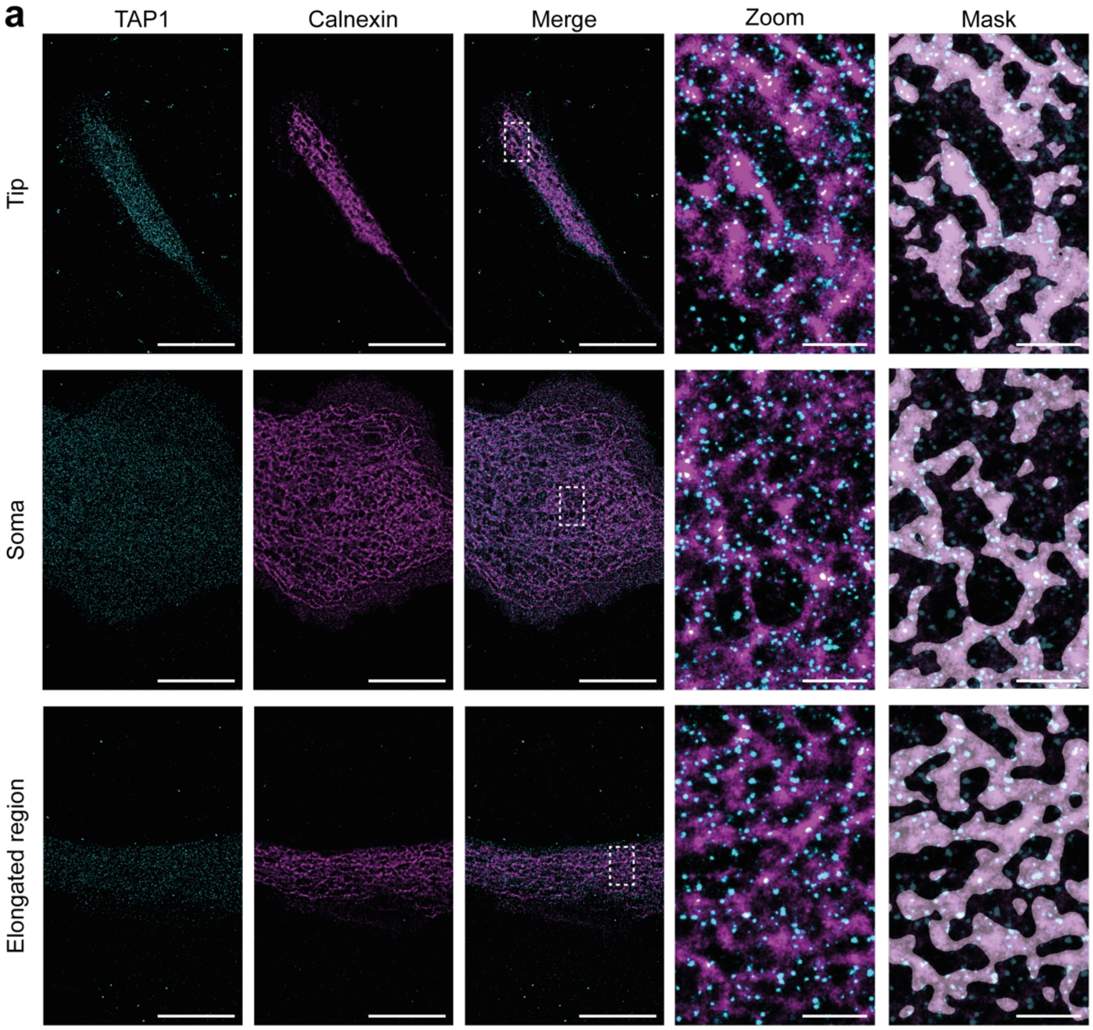

a
TAP1
Calnexin
Merge
Zoom
Mask
Tip
Soma
Elongated region

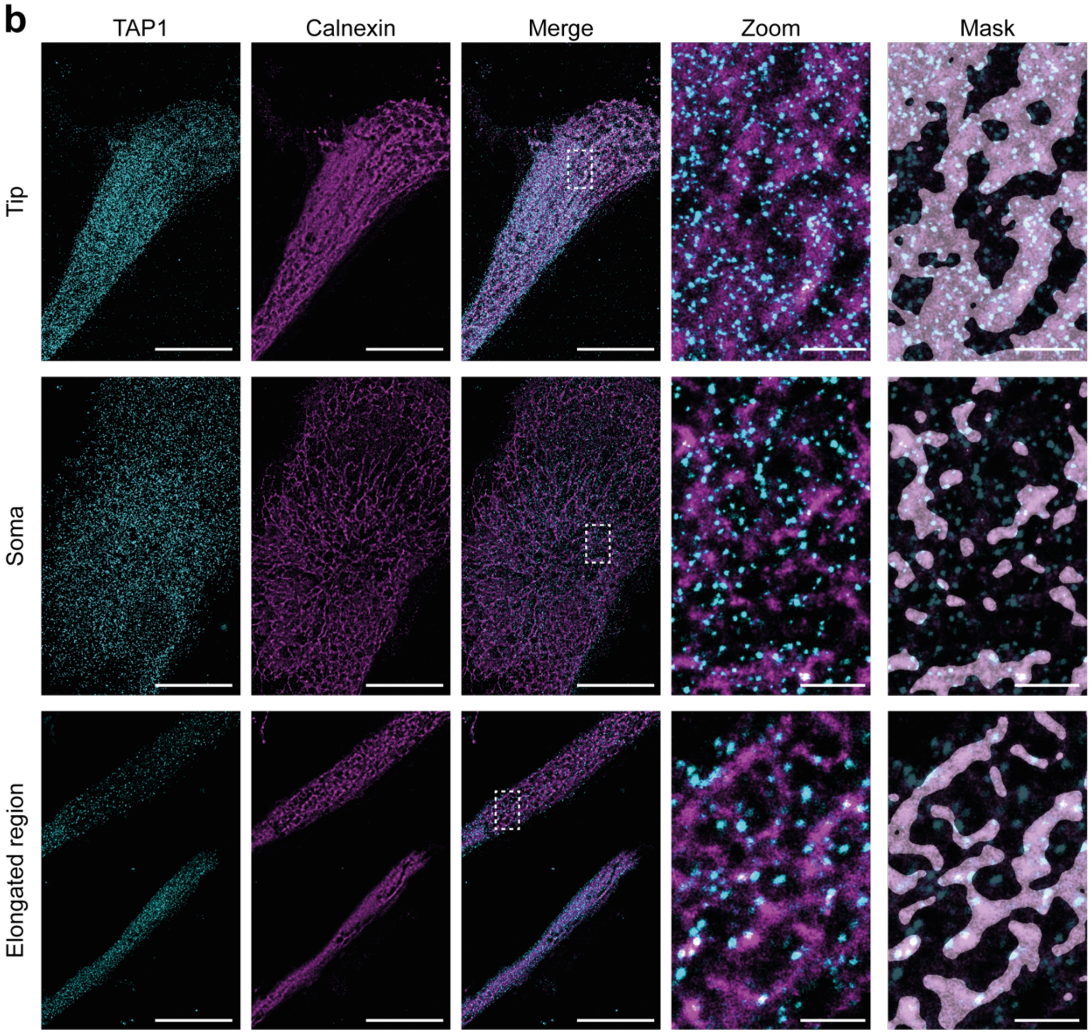
b
TAP1
Calnexin
Merge
Zoom
Mask
Tip
Soma
Elongated region

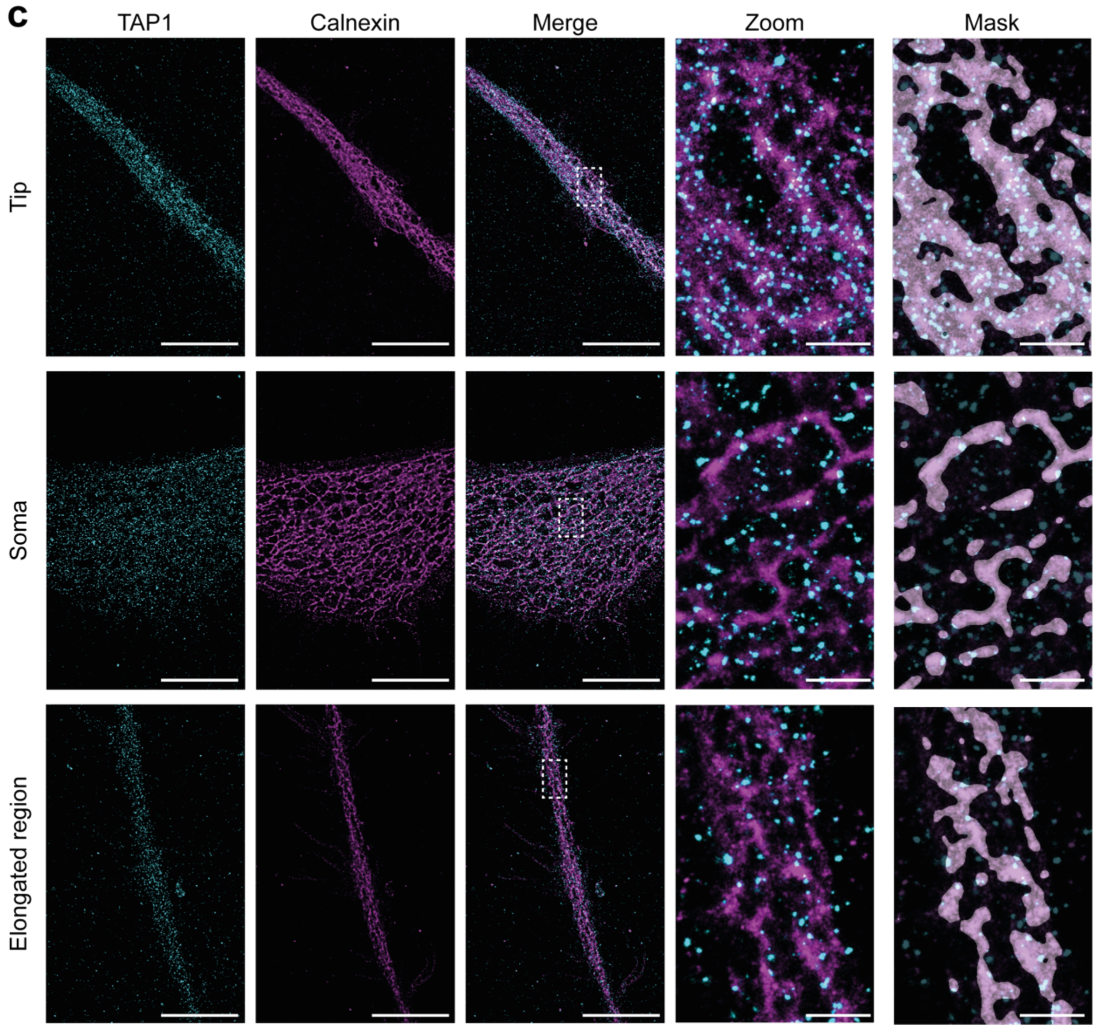

Supplementary Fig. 4. Calnexin and TAP1 colocalize to different degrees in mDCs. Reconstructed super-resolved images of fixed mDCs. Cells were treated with 3% (v/v) formaldehyde and 0.1% (v/v) glutaraldehyde for 1 h and subsequentially labeled with the corresponding primary and secondary antibodies (AF532 for calnexin and AF647 for TAP1). Both channels were aligned with 100 nm Tetraspecks as fiducial markers. TAP1 is depicted in cyan and calnexin in magenta. Panel **a**, **b**, and **c** display representative examples of mDCs derived from three different donors with focus on the tip of a protrusion (top panel), soma area (middle panel), and the elongated region between soma and tip (bottom panel). The dashed white boxes indicate the region of interest of the zoom. All nine exemplary images are comparable in co-localization behavior for TAP1 and calnexin. Tip regions typically show a high density of TAP1 and calnexin with TAP1 following mostly the ER-leaflet structures in close proximity to the ER-marker calnexin. The soma region exhibits well-resolved ER structures whereas in the tip region the densely packed ER structure exhibits a condensed signal accumulation. However, only a few directly co-localized spots are visible. The elongated regions typically exhibit a stretched ER structure with comparable TAP1 to calnexin ratio as in the soma area. For a better visual guidance, for each zoom the filter mask was artificially created in ImageJ using the calnexin only signal.

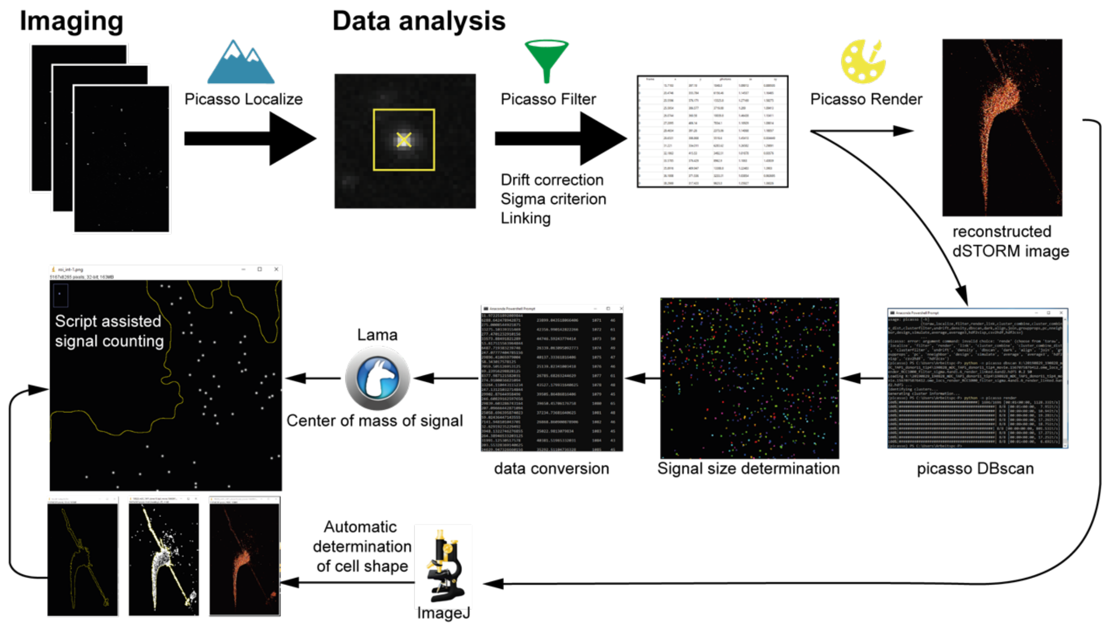

Supplementary Fig. 5. Extended imaging and analysis workflow overview. Single-color experiments were performed as follows: Imaging of 30,000 frames with 30 ms integration time and without 405 nm excitation for 10,000 frames, followed by a gradual increase of 405 nm reactivation laser every 5,000 frames for 0.5% to a final value of 2%. This gradient ensured a suitable emitter density for the whole imaging time. The Picasso plugin Localize was used to determine the center of each point spread function. Afterwards, drift correction as well as filtering and linking of single-molecule emission events were performed. The localization data was subsequentially rendered with Picasso Render generating a high-resolution image. This image was analyzed in ImageJ using a home-written script to determine cell outlines. Localization data were further analyzed for clustering of signals using the DBSCAN algorithm implemented in Picasso. Subsequently, an additional script was used to convert the DBSCAN file into the format required for the software LAMA. LAMA creates an image in which the center of mass of individual signals is assigned by one gray value in its respective pixel of localization, resulting in an image that allows counting in specific regions of interest. The previously generated cell outline was used to measure the integrated intensity in the signal image, which provides the number of signals in this region. In the end the exact number of signals as well as the cell area were determined and used for the density calculations

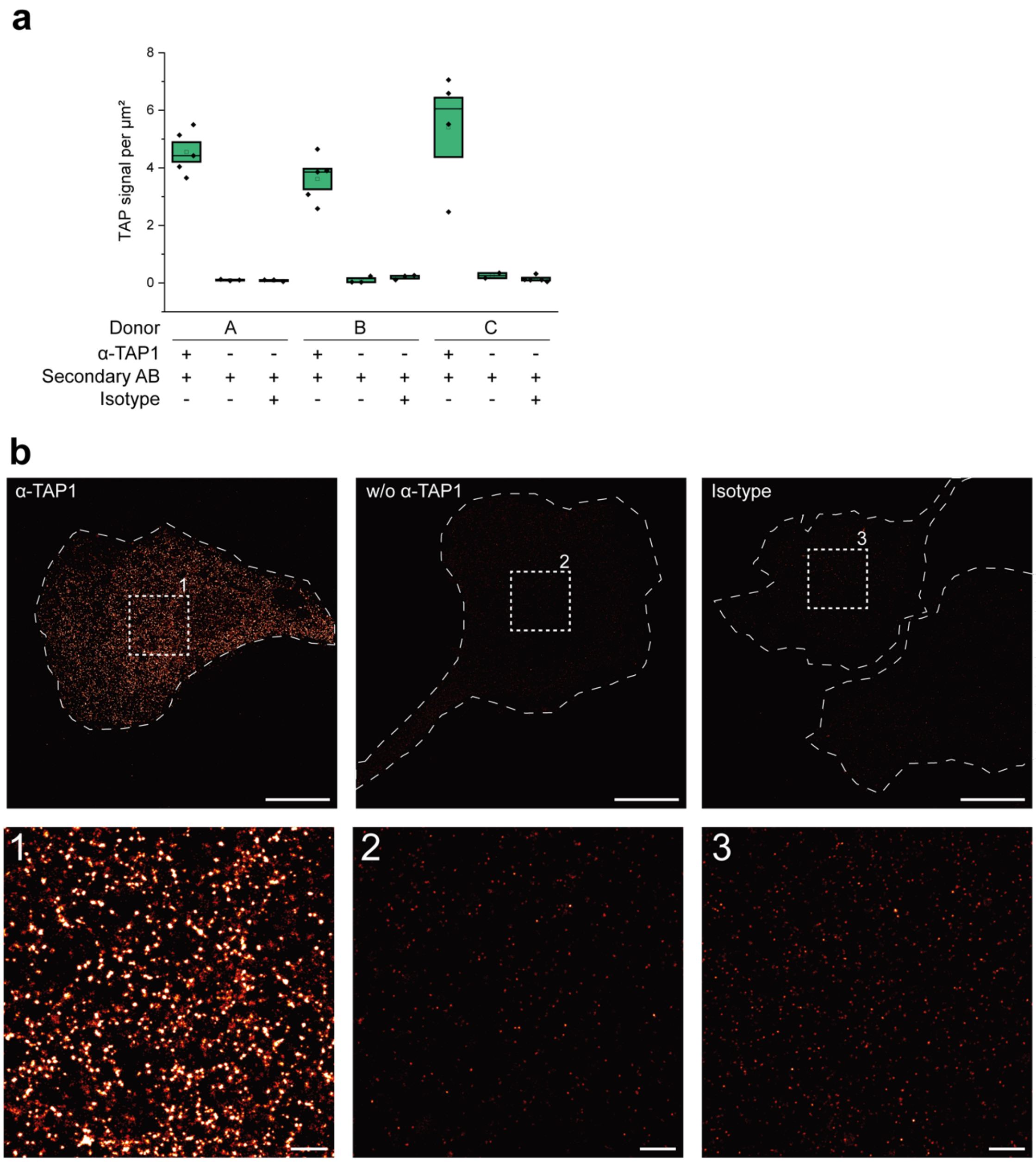

Supplementary Fig. 6. Labeling of TAP1 with α-TAP1 antibody, without antibody and with an isotype antibody. The specificity of the α-TAP1 antibody staining was verified by comparing the signals per area in moDCs with α-TAP1 antibody, secondary antibody only, and with an isotype antibody. These experiments were treated and analyzed as described before. **a** Whisker plot for three donors (A-C) with each five cells imaged. The α-TAP1 antibody staining yielded around 4.5 ± 0.8 signals/µm² whereas isotype antibody and secondary antibody only led to ~0.15 ± 0.06 signals/µm² verifying the lack of unspecific background signal. **b** The top panel shows an exemplary overview for all three conditions with α-TAP1 antibody, without the latter and with the isotype antibody only. Cell outline is emphasized with dashed lines, and dashed boxes indicate regions that are shown as zoom-ins (bottom). Scale bars are 10 µm for overview image and 1 µm for zoom-ins.

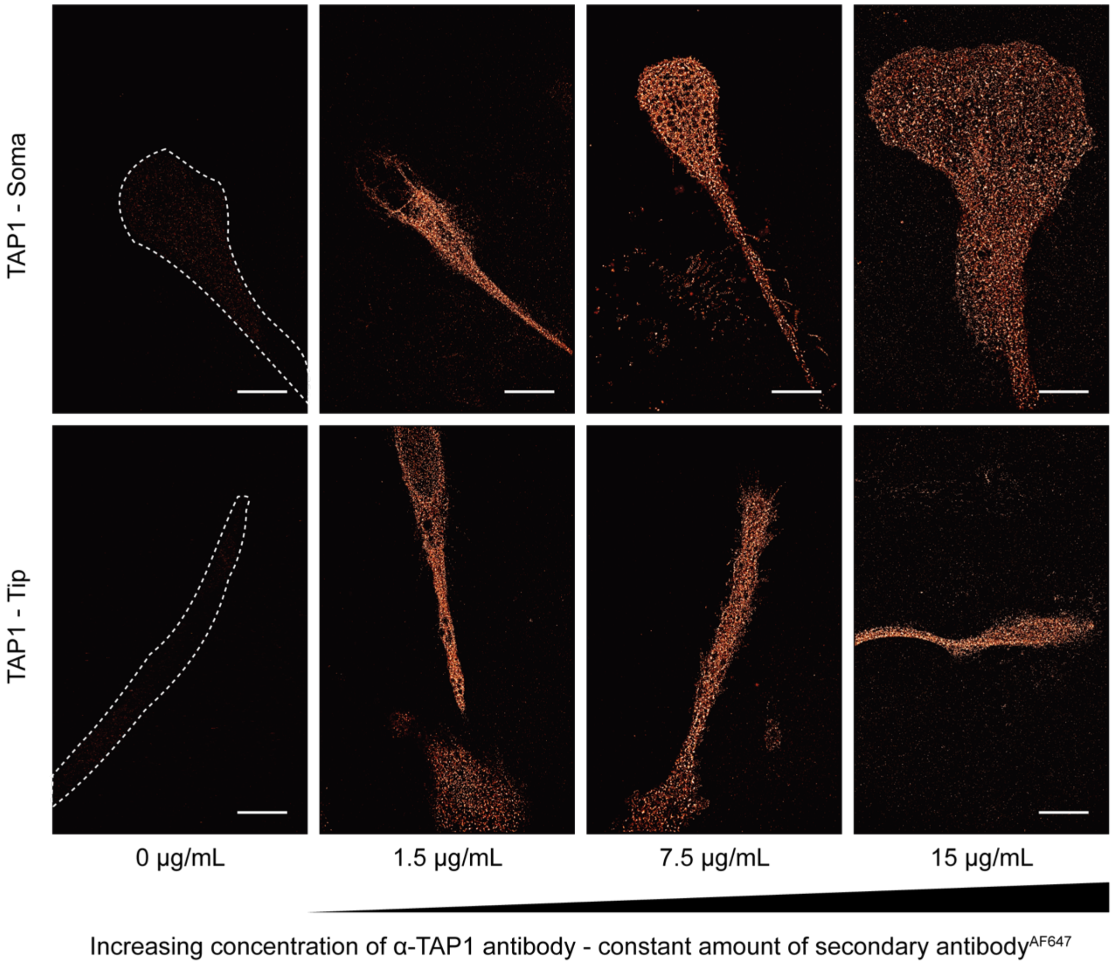

Supplementary Fig. 7. Titration of α-TAP1 with constant secondary antibody labeled with AF647. To ensure maximal labeling efficiency with minimal unspecific staining of moDCs, the labeling of TAP1 was analyzed with increasing concentrations of α-TAP1 antibody ranging from 0 to 15 µg/mL. The concentration of the secondary antibody carrying the fluorophore was kept at a 1:100 dilution (final 20 µg/mL) as described in the methods section. The top panel shows an insight into TAP distribution over the soma area and the bottom panel of the tip part of mDC protrusions. Scale bars are 10 µm. Without α-TAP1 addition only background fluorescence is visible, and the intensity of the signals remain constant with increasing amounts of primary antibody up to the final concentration of 15 µg/mL. Correspondingly, the concentration of 1.5 µg/mL of α-TAP1 antibody was used for the experiments.

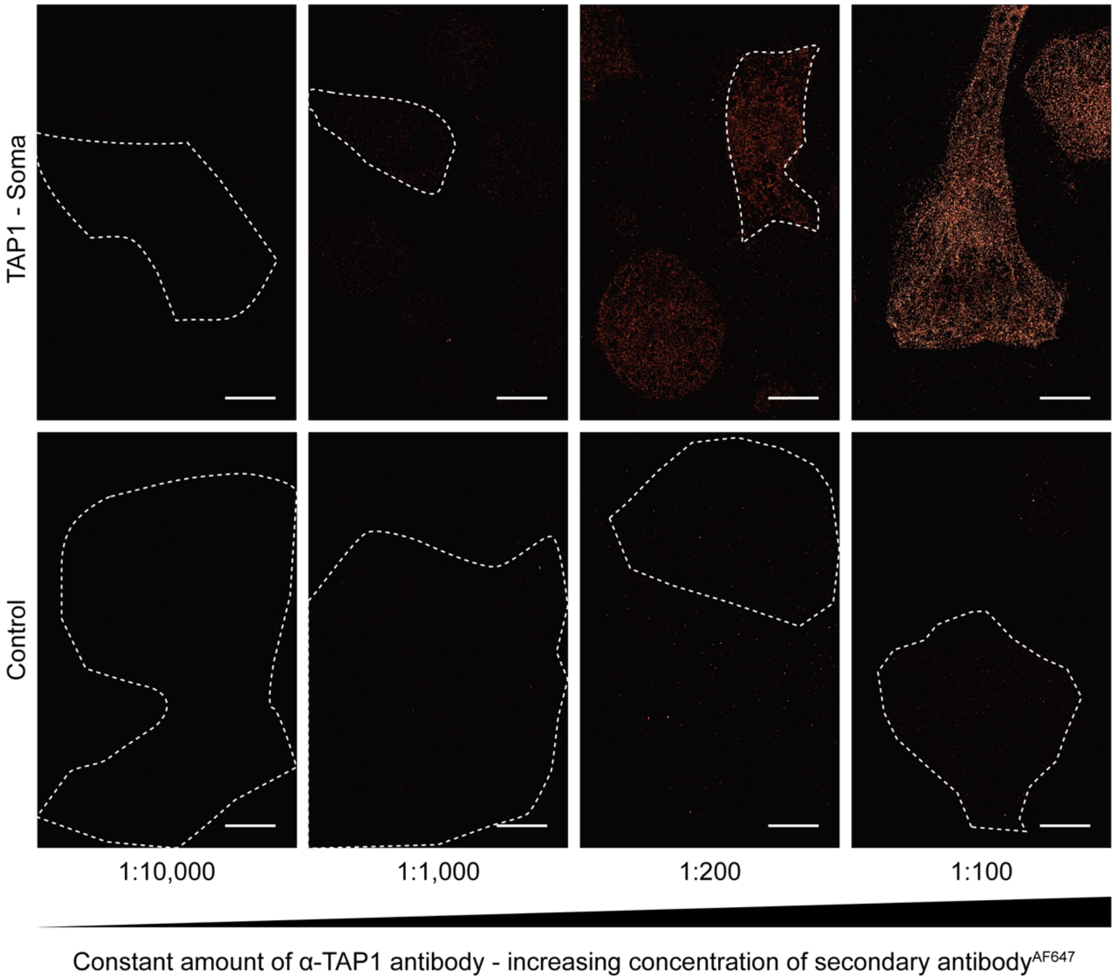

Supplementary Fig. 8. Titration of secondary antibody labeled with AF647 with constant amount of α-TAP1 antibody. To determine the optimal staining efficiency of the secondary antibody, a fixed concentration of 1.5 µg/mL of α-TAP1 was used and the concentration of the secondary antibody was varied between a 1:10,000 and a 1:100 dilution. A dilution of 1:10,000, 1:1,000, and 1:200 resulted in no or weak staining. 1:100 dilution (final 20 µg/mL) provided the best staining results with still low unspecific staining in the control. This antibody concentration was thus used for all super-resolution experiments. Scale bars are 10 µm.

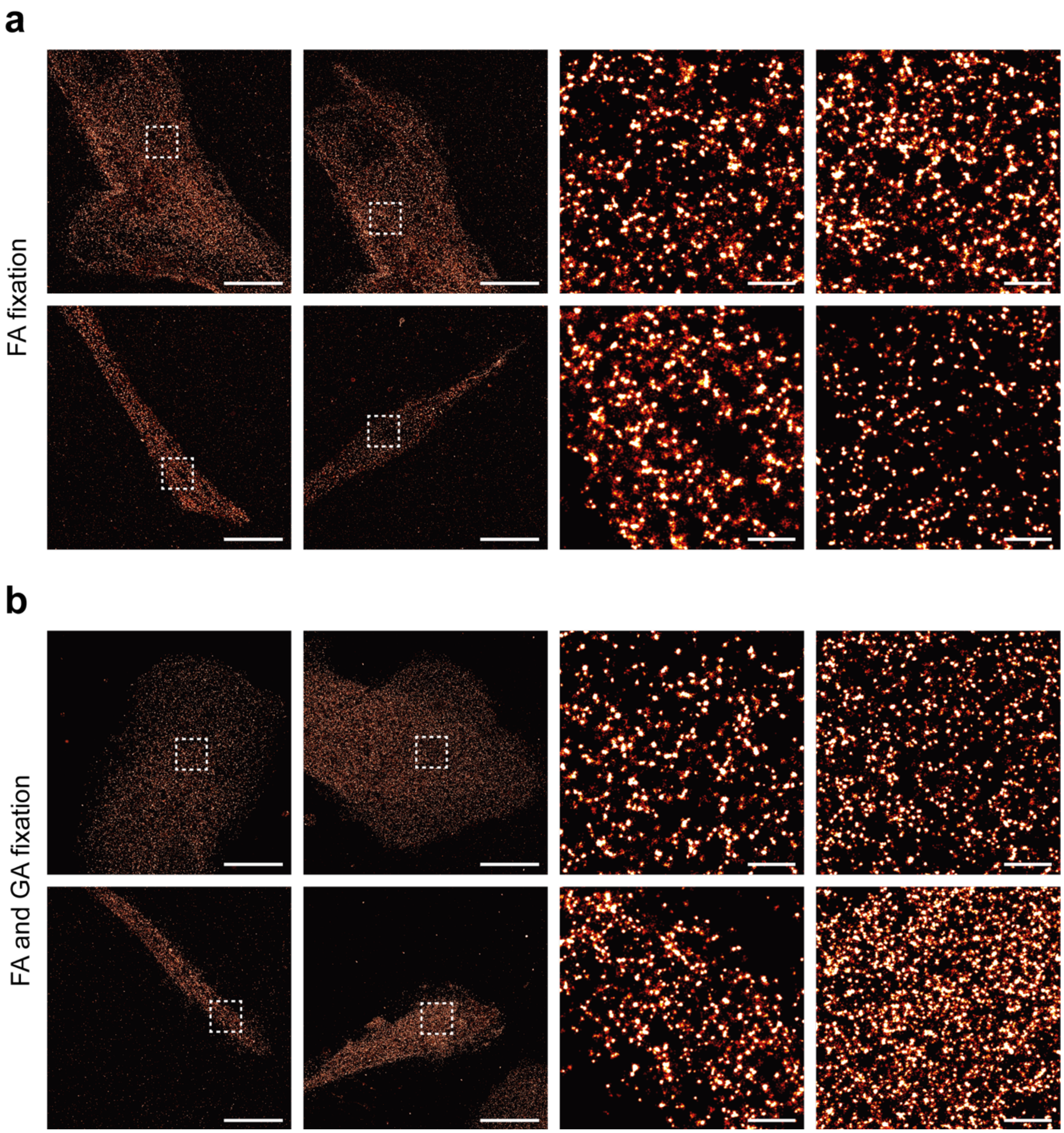

Supplementary Fig. 9. Comparative analysis of fixation reagents. Comparison of fixation methods with 3% (v/v) formaldehyde diluted in PBS versus 3% (v/v) formaldehyde and 0.1% (v/v) glutaraldehyde diluted in PHEM buffer (60 mM PIPES, 25 mM HEPES, 10 mM EGTA, 4 mM $MgSO_4$, adjusted to pH 6.9 with KOH). Cell overview (scale bar 10 µm) and dashed white lines with zoom-ins (scale bar 1 µm) of exemplary TAP signals. **a** Formaldehyde fixation depicts individual separated signals. Upper panel with two example cells on the left and associated zoom-ins from dashed white boxes. **b** Formaldehyde/glutaraldehyde fixed cells do not exhibit apparent differences in size and distribution of TAP signal as compared to moDCs fixed with formaldehyde only. Upper panel with focus on soma area and bottom panel with tip area. Adjacent zoom-ins depict area of white dashed boxes.
